# Reactive Sulfur Species and Protein Persulfidation: An Emerging Redox Axis in Human Health and Disease

**DOI:** 10.3390/cimb47090765

**Published:** 2025-09-16

**Authors:** Celia María Curieses Andrés, Fernando Lobo, José Manuel Pérez de la Lastra, Elena Bustamante Munguira, Celia Andrés Juan, Eduardo Pérez Lebeña

**Affiliations:** 1 Hospital Clínico Universitario de Valladolid, Avenida de Ramón y Cajal, 3, 47003 Valladolid, Spain; cmcuriesesa@saludcastillayleon.es (C.M.C.A.); ebustamante@saludcastillayleon.es (E.B.M.); 2 Institute of Natural Products and Agrobiology, CSIC-Spanish Research Council, Avda. Astrofísico Fco. Sánchez, 3, 38206 La Laguna, Spain; fernando.lobo@csic.es; 3 Department of Organic Chemistry, Cinquima Institute, Faculty of Sciences, Valladolid University, Paseo de Belén, 7, 47011 Valladolid, Spain; 4 Sistemas de Biotecnología y Recursos Naturales, 47625 Valladolid, Spain; info@glize.eu

**Keywords:** reactive sulfur species, hydrogen sulfide, persulfidation, trans-sulfuration pathway, redox signaling, NRF2/Keap1, H_2_S donors, chemoproteomics, cardiovascular and metabolic disease

## Abstract

Reactive sulfur species (RSS)—hydrogen sulfide (H_2_S), low-molecular-weight persulfides/polysulfides and protein persulfidation—constitute a third redox axis alongside ROS and RNS. Nanomolar H_2_S, produced by trans-sulfuration (CBS/CSE) and 3-MST, is oxidized by sulfide–quinone reductase to persulfides that fuel the respiratory chain while curbing superoxide. Reversible persulfidation reprograms cysteine sensors in metabolism (GAPDH), inflammation (NLRP3, p47^phox^) and transcription (Keap1/NRF2), linking RSS to energy balance, vasodilation, innate immunity and neuroplasticity. Disrupted sulfur signaling—deficit or overload—contributes to heart failure, sarcopenia, neurodegeneration, cancer and post-COVID syndromes. Therapeutically, slow-release donors (SG1002, GYY4137), mitochondria-targeted vectors (AP39), photo- or thiol-activated “smart” scaffolds, diet-derived polysulfides/isothiocyanates and microbiota engineering aim to restore the protective RSS window. Key challenges are a narrow therapeutic margin and real-time quantification of persulfide fluxes. Harnessing RSS therefore offers a route to rebalance redox homeostasis across diverse chronic diseases.

## 1. Introduction

During the last four decades, the study of reactive species has become one of the backbones of redox biology. After the discovery of reactive oxygen species (ROS) as metabolic by-products that could, depending on the context, damage or signal—a concept consolidated in the 80s with the definition of “oxidative stress” by Helmut Sies—[1] other chemically analogous families appeared but with different central atoms: reactive nitrogen species (RNS), [2] reactive halogen species (RHS) and, more recently, the reactive sulfur species (RSS) [3]. Each group shares the ability to transfer an electron or an atom to biomolecules, but differs in their fundamental chemistry, kinetics and cellular targets.

Under the umbrella of RSS, sulfur-containing molecules are grouped into different oxidation states and with sufficient thermodynamic instability to react with nucleophiles or biological electrophiles: (i) hydrogen sulfide (H_2_S) and its anions at physiological pH, (ii) persulfides and polysulfides of low molecular mass (RSSH, RSS^−^, H_2_Sn), and (iii) protein persulfation (R-S-SH), now accepted as a new redox signaling code [4,5,6]. These species are generated endogenously through the enzymes of the trans-sulfuration pathway—cystathionine-β-synthase (CBS) and cystathionine-γ-lyase (CSE)—and 3-mercaptopiruvate sulfurtransferase (MPST/3-MST), as well as by mitochondrial sulfur oxidation pathways; exogenously, they come from sulfur dietary compounds and pharmacological donors designed to release H_2_S or polysulfides [7,8]. Unlike ROS or RNS, the sulfur atom can oscillate between oxidation states −2 and +6, which gives RSS a unique chemical plasticity and the ability to self-react, for example when sulfide reduces protein sulfenyls and is converted to persulfide [9,10].

Table 1 summarizes the main characteristics of the different reactive species.

Since Helmut Sies coined the term “oxidative stress” in 1985 to describe the cellular imbalance caused by reactive oxygen species (ROS), [1] the field of redox biology expanded rapidly: in 1991, Salvador Moncada and collaborators demonstrated that nitric oxide acted as an endogenous vasoregulator, inaugurating the family of reactive nitrogen species (RNS) [11]; five years later, Hideo Kimura identified the brain’s production of H_2_S, laying the foundation for RSS [12]; the global notion of RSS was consolidated in 2017 with the review “The Reactive Sulfur Species Concept: 15 Years On”, which underlined the physiological relevance of protein persulfidation [13]; in the 2020s, click-chemistry probes made it possible to map persulfidations on a large scale and, in parallel, clinical donors such as SG1002 moved to phase II (NCT02278276) trials, placing reactive sulfur at the forefront of redox pharmacology.

**Figure 1:**
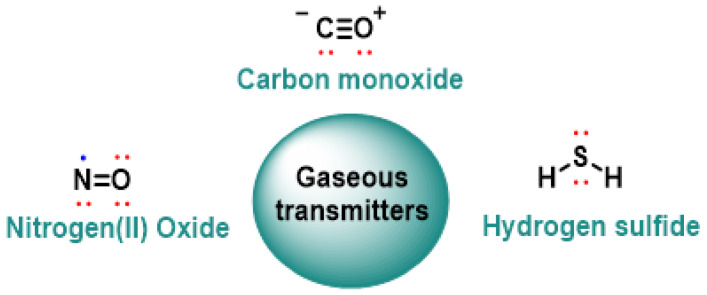
The three gaseous signaling molecules involved in biological functions.

Thus, RSS completes the panorama of reactive species by providing chemical versatility, reversible bonds and metabolic pathways closely linked to nutrition and the microbiota. Understanding it is essential to decipher how sulfur acts as the third pillar of redox signaling in health and disease, (Figure 1) [13].

The acceptance of RSS as a bona fide third redox axis has progressed through several debates and turning points. First, early claims of high intracellular H_2_S conflicted with later work showing rapid mitochondrial oxidation via SQR/ETHE1 and much lower steady-state levels (nanomolar in brain, low micromolar in aorta), reframing H_2_S as a tightly regulated messenger rather than a bulk reductant. These physiological ranges, together with the genetic evidence that defects in sulfide oxidation (e.g., ETHE1) cause severe mitochondrial disease, were pivotal for credibility. Second, the field moved from the modified biotin-switch to improved tag-switch and quantitative click chemistries, alongside calibrated sulfane–sulfur probes (e.g., SSP4), and the discovery that TRX/GRX systems act as depersulfidases. Collectively, these tools addressed concerns about artifacts and established protein persulfidation as a reversible, enzyme-linked signal rather than a chemical curiosity. Third, the identification of mixed NO–sulfur intermediates (HSNO/SSNO^−^) as potential “relays” in NO–H_2_S crosstalk offered a mechanistic bridge between axes; although supportive chemical and physiological data exist, questions remain about their lifetimes and compartmentalization in vivo. Finally, translation advanced when slow-release H_2_S donors entered clinical testing (e.g., SG1002), signaling therapeutic plausibility even as dose, release kinetics and off-target effects continue to be refined. Throughout, lingering uncertainties in quantitative speciation (free H_2_S vs. polysulfides vs. protein-SSH) and inter-laboratory variance—summarized in Section 3.1—explain divergent effect sizes and remain active areas for standardization [14,15,16,17,18].

Overall, the purpose of this article is to offer a panoramic and critical view of the “sulfur axis” of redox biology: (i) to describe the chemistry and biosynthetic pathways that give rise to sulfide, persulfides and polysulfides; (ii) explain how protein persulfidation modulates metabolic, inflammatory and epigenetic pathways in dialogue with ROS and RNS; (iii) to evaluate the preclinical and clinical evidence that positions H_2_S donors and sulfur dietary compounds as therapeutic strategies; and (iv) point out the analytical challenges, knowledge gaps and research priorities that need to be addressed in order to translate these findings into next-generation nutritional and pharmacological interventions. In this way, we intend to consolidate the conceptual framework of RSS, integrate its relevance in health and disease and stimulate new interdisciplinary lines of work that take advantage of the regulatory potential of reactive sulfur.

To help unify the diverse topics covered in this review, we frame the “RSS axis” as a simple left-to-right flow: endogenous generation → chemical speciation → reactivity mode → biological readouts. First, endogenous H_2_S/RSS production by CBS, CSE and MQO establishes the basal sulfur flux within cells. Second, this flux is funneled into persulfides and polysulfides, creating a dynamic pool in which chain length and local environment tune stability and transfer potential. Third, the resulting species express a bimodal chemical personality: nucleophilic behavior (e.g., HS^−^/RSS^−^ acting as soft bases) versus electrophilic behavior (e.g., S_n_ species transferring S^0^) that enables selective persulfidation of cysteine residues and other sulfur-exchange reactions. This textual model—sources feeding polysulfide formation, which in turn toggles between nucleophilic and electrophilic chemistries—provides the narrative spine for the sections that follow, clarifying how enzyme-level control (CBS/CSE/MQO) sets the stage for polysulfide biogenesis and, ultimately, for context-dependent RSS signaling through reversible sulfur transfer.

## 2. Chemistry of RSS. Endogenous Generation (CBS/CSE, MQO), Polysulfide Formation and Electrophilic/Nucleophilic Characteristics

In mammals, the chemical backbone of RSS begins with endogenous production of H_2_S. Three enzymes in the trans-sulfidation pathway—cystathionine-β synthase (CBS), cystathionine-γ-lyase (CSE) and 3-mercaptopiruvate sulfurtransferase (3-MST, sometimes referred to as MPST or MQO)—constantly generate this gas transmitter from homocysteine, cysteine or 3-mercaptopiruvate, respectively [19].

**Figure 2:**
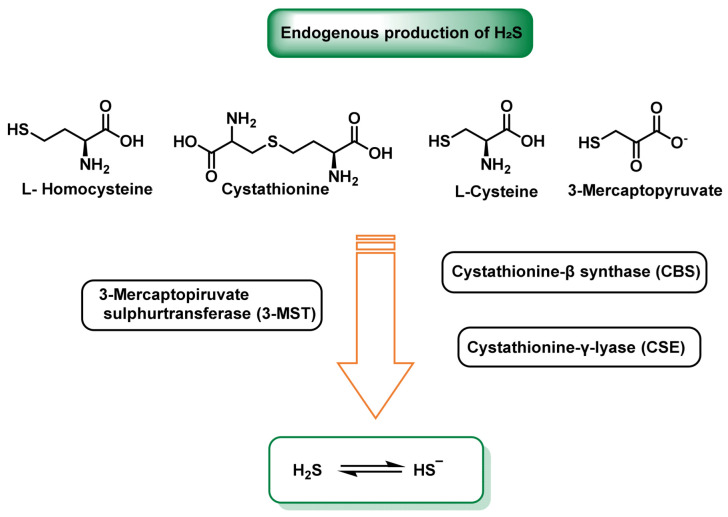
The biosynthesis of H_2_S mammalian cells primarily involves cystathionine β-synthase (CBS), cystathionine-γ-lyase (CSE) and 3-mercaptopyruvate sulfurtransferase (3MST).

Cystathionine-β-synthase, cystathionine-γ-lyase and mercaptopyruvatesulfur transferase are part of the transsulfuration pathway, the latter involved in cysteine catabolism (Figure 2).

**Figure 3:**
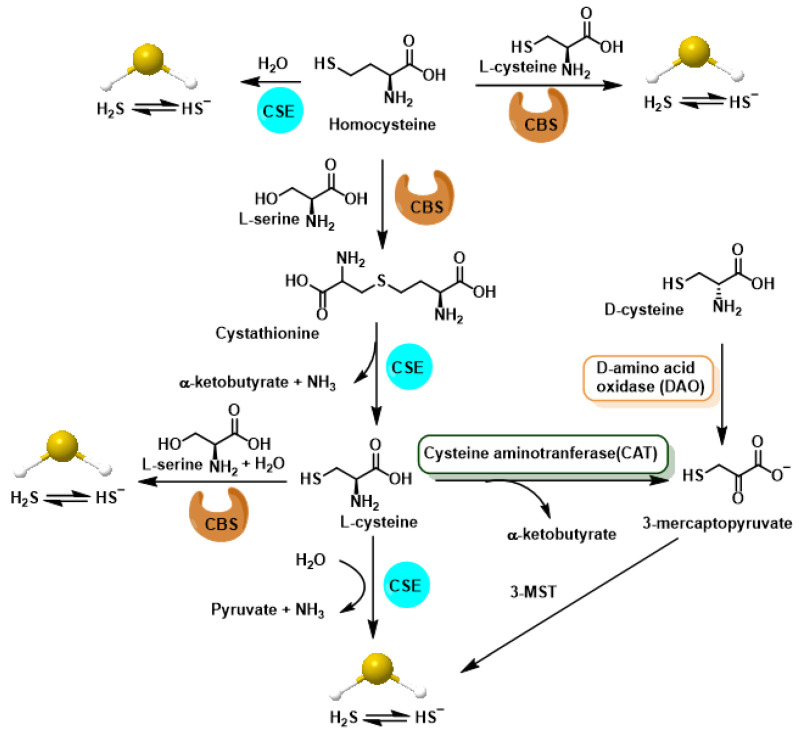
Routes of H_2_S synthesis mediated by CBS, CSE and 3MST enzymes.

The H_2_S synthesis pathways mediated by CBS, CSE and 3-MST enzymes are shown in Figure 3.

The tissue and subcellular distribution of H_2_S-producing enzymes gives the sulfide system fine-tuned control over its active levels. Cystathionine-β-synthase (CBS) is robustly expressed in the brain and liver, where it becomes the main source of H_2_S and, consequently, of neuronal and hepatic protein persulfidation, to the point that its deficiency precipitates neurometabolic alterations and liver diseases [20,21]. On the other hand, cystathionine-γ-lyase (CSE) dominates in the endothelium and vascular smooth muscle; there its transcription increases with laminar flow and its activity determines the bioavailability of H_2_S that modulates vasorelaxation and the inflammatory response [22,23]. The third enzyme, 3-mercaptopiruvate sulfurtransferase (3-MST), is distributed between cytosol and mitochondrial matrix of practically all organs, supplying sulfur directly to respiratory complex III and acting as a persulfidase on target proteins, which ensures local signaling even in cells with low expression of CBS or CSE [24].

This anatomical specialization and rapid mitochondrial catabolism via sulfur-quinone–oxidoreductase (SQR) keep H_2_S free in the range of 10–15 nM in the brain and about 1 μM in the aorta—values three orders of magnitude lower than those that alter cell function in in vitro experiments [14,25]. In fact, concentrations that produce acute toxic symptoms in humans (≈20 ppm, ~28 μM dissolved) far exceed the physiological threshold, underscoring the need for strict enzyme regulation to take advantage of the gas’s signaling properties without triggering necrosis or respiratory inhibition (https://www.osha.gov/hydrogen-sulfide/hazards, accessed on 2 September 2025). Thus, the differential expression of CBS, CSE and 3-MST, together with oxidative catabolism and mitochondrial compartmentalization, ensures that sulfide acts as a reversible messenger at nano-micromolar concentrations, without being cytotoxic.

Once released by trans-sulfuration enzymes, the HS^−^ anion is not loose in the cytosol: it is almost immediately “fished” by the sulfide–quinone–oxidoreductase (SQR) embedded in the mitochondrial inner membrane. SQR extracts two electrons from the sulfide and injects them into the ubiquinone of the respiratory chain while forming a covalent persulfide over its own FAD center. This “activated” sulfur is then transferred by transpersulfidation to glutathione to give rise to glutathione–persulfur (GSSH), or passes to rhodanese (TST) or persulfur–dioxygenase ETHE1. In these divergent pathways, the rhodanese fuses the sulfur with another sulfite or polysulfide residue to yield thiosulfate, while ETHE1 oxidizes the GSSH to sulfite, which continues to sulfate by sulfite oxidase [26,27]. Part of the persulfide generated by SQR, however, escapes this metabolization and spontaneously condenses with another HS^−^, giving H_2_Sn linear chains or Sn^2−^ anions that diffuse into the cytosol and function as a reservoir of “sulfur sulfan”. This abiotic process is accelerated when mild oxidants such as molecular oxygen, hydrogen peroxide or chloric hypochlorite intercept HS^−^ and facilitate the formation of polysulfides [28,29]. The integrity of the pathway is decisive: mutations that inactivate SQR or ETHE1 trigger tissue accumulation of sulfide, destabilize polysulfides and block mitochondrial respiration, a condition that in humans manifests as infantile ethylmalonic encephalopathy, while in animal models it causes bioenergetic failure and cell death [30]. These findings underscore that sequential oxidation of sulfide—from HS^−^ to persulfide, thiosulfate and sulfite—not only prevents its toxicity, but preserves a dynamic “pool” of polysulfide ready to be converted back into a redox signal or shield as dictated by the physiological state of the cell.

**Figure 4:**
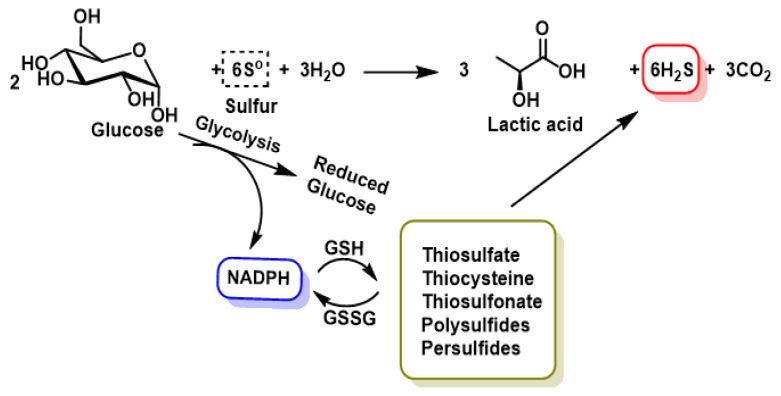
Non-enzymatic routes of H_2_S synthesis. In the presence of reducing equivalents such as NADPH and NADH, reactive sulfur species in persulfides, thiosulfate and polysulfides are reduced into H_2_S and other metabolites. GSH is glutathione and GSSG is glutathione disulfide.

Non-enzymatic H_2_S production, although less important, occurs via glucose, glutathione, organic and inorganic polysulfides and elemental sulfur. The reduction of elemental sulfur using NADPH or the oxidation of glucose in the phosphogluconate pathway also produces H_2_S in a non-enzymatic form. The reduction of elemental sulfur to H_2_S using reductants from glucose oxidation, such as lactate, or others such as nicotinamide adenine dinucleotide hydrogen (NADH), nicotinamide adenine dinucleotide phosphate (NADPH) and glutathione (Figure 4).

**Figure 5:**
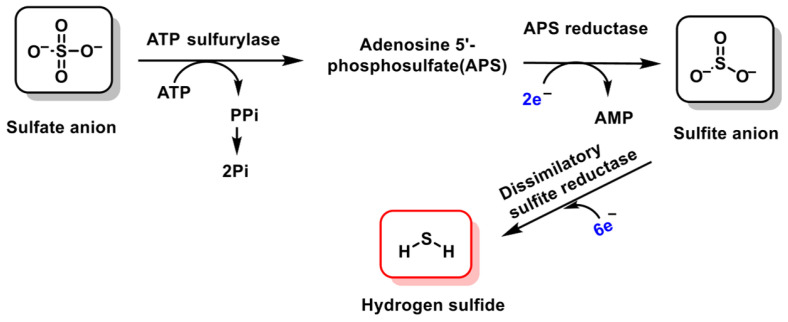
Dissimilatory sulfate reduction pathway.

Intestinal bacteria generate H_2_S by using sulfate as their terminal electron acceptor, adding to the baseline H_2_S produced by host CBS and CSE enzymes in the gut lining. As a result, the digestive tract—particularly the intestinal epithelium—is exposed to substantial quantities of this gas. The levels of microbially derived H_2_S, however, fluctuate depending on dietary substrates and the composition of the gut microbiota. Beyond its synthesis via cysteine catabolism, H_2_S also arises through the dissimilatory reduction of dietary sulfate, (Figure 5), underscoring its significant impact on gastrointestinal health [31,32].

The unique reactivity of RSS stems from the ability of sulfur to behave as both a soft nucleophile and a soft electrophile. In its ionized form, HS^−^ (pK_a_≈7.0) is already a more potent nucleophile than conventional thiolates, but when sulfur forms a persulfide (RSS^−^) the acidity of the S–H bond drops to pK_a_ 5.5–6.5; at physiological pH, the species is almost completely deprotonated, which raises the electron density above the terminal atom and accelerates carbonyl reactions by several orders of magnitude: α,β-unsaturated, with hydroxyl or peroxyl radicals and with metals such as Cu(I) or Hg(II). In this way, persulfides act simultaneously as radical terminators and as selective chelators, providing a double line of antioxidant defense [33,34,35].

That same electron density is reversed in the inner atom of the –S–S–H bond, which exhibits a positive partial-charged “σ-hole” capable of accepting electron pairs from other thiolates or selenolates; the resulting electrophilia drives the trans-persulfidation reactions by which sulfur is transferred to regulatory cysteines. Thus, the persulfidation of Keap1 in Cys151 causes the release of NRF2 and activates the antioxidant response, while that of glyceraldehyde 3-phosphate dehydrogenase (GAPDH) in Cys156 attenuates its glycolytic activity and redirects the flow of carbon under oxidative stress [36,37,38]. GAPDH is an enzyme that catalyzes the sixth step of glycolysis and thus serves to break down glucose for energy and carbon molecules.

The modification is fully reversible thanks to the thioredoxin and glutaredoxin pathways, which reduce both protein persulfides and polysulfides and regenerate H_2_S; this “compensation chamber” keeps the free sulfide below the toxic threshold and allows the signals to turn on and off in a matter of seconds or minutes, providing the cell with an extraordinarily plastic redox regulator. The ease with which sulfur oscillates between oxidation states −2 and +6 ultimately explains why RSS can act both as antioxidant buffers and as reversible messengers that coordinate metabolism, inflammation and cell death [39,40].

### Quantitative Kinetics: Persulfide Formation Versus Oxidation

To complement the qualitative description above, we summarize benchmark second-order rate constants (k_2_) under near-physiological conditions. These values make explicit the kinetic competition between (i) persulfide formation (e.g., HS^−^ attack on protein sulfenic acids or sulfur transfer from low-molecular-weight persulfides) and (ii) oxidation of H_2_S/HS^−^ by common oxidants. Stopped-flow studies indicate that HS^−^ attacks protein—SOH with k_2_ ≈ 1.4 × 10^3^ M^−1^ s^−1^ (Mt AhpE–SOH model), while hydrophilic persulfides modify protein cysteines with k_2_ in the 10^4^–10^5^ M^−1^ s^−1^ range—two to three orders of magnitude faster than classical Michael electrophiles (≈10^2^ M^−1^ s^−1^), Table 2. In parallel, oxidation of H_2_S/HS^−^ proceeds slowly with H_2_O_2_ but much faster with peroxynitrous acid and especially HOCl; chloramines react more slowly but with higher selectivity. Where HOCl/ONOOH are abundant (e.g., inflamed niches), oxidation can outcompete persulfidation and drain H_2_S into more oxidized products; conversely, in mild oxidant settings, rapid persulfidation can divert –SOH away from over-oxidation and maintain reversible sulfur signaling. 

## 3. Protein Persulfidation. Mechanisms, Detection Techniques, Comparison with Nitrosylation and Glutathionylation

Persulfidation, the addition of a sulfur sulfan atom that converts a thiol group (–SH) to a persulfide (–SSH), is a ubiquitous post-translational modification with an impact comparable to that of nitrosylation or glutathionylation. Unlike classical cysteine oxidations, the new S–S–H bond retains complete reversibility and can remodel pre-existing disulfides or sulfenyls, modulating processes as diverse as energy metabolism, inflammation or the response to oxidative stress [41,42].

Mechanistically, the additional sulfur comes mainly from endogenous H_2_S and, more efficiently, from polysulfides (H_2_Sn, Sn^2−^). These species transfer “activated sulfur” to cysteines that are in electrophilic states (–SOH), nitrosothiol (–SNO) or disulfide—or when the protein is attacked by HS^•^ radicals formed in the presence of transition metals. The resulting persulfide, of pK_a_≈5.5–6.5, is much more nucleophilic than a conventional thiol and can propagate the mark by trans-persulfidation; thus, the modification of Cys151 in Keap1 activates the NRF2 pathway, and that of Cys156 in GAPDH redirects the glycolytic flow [37,43,44].

Far from being a permanent seal, persulfidation is fully reversible: the thioredoxin and glutaredoxin systems reduce –SSH and regenerate H_2_S with kinetics higher than those shown against disulfides, ensuring that the signal oscillates between −2 and 0 states without producing irreversible oxidative damage [16,40].

The experimental take-off of “persulfidomics” was supported by analytical advances. The modified-biotin switch gave way to the tag-switch, in which the persulfide is blocked with methylsulfonyl and then developed with azides or alkynes for click capture; this strategy supports quantitative proteomics protocols of site-specific resolution [45]. In parallel, activatable fluorescent probes such as SSP4 allow the dynamics of sulfans to be followed in vivo, and iodoacetamide–alkyne derivatives, in isotopic versions, facilitate quantitative comparisons between physiological and pathological conditions [46,47].

Recent chemoproteomic maps that leverage improved tag-switch workflows for selective capture of –SSH, complemented by cysteine-reactivity profiling with isotopically labeled iodoacetamide–alkyne probes and sulfane–sulfur tracers, converge on a conserved “core persulfidome.” Across human and murine cells, high-confidence –SSH calls cluster in central carbon metabolism—most prominently GAPDH and other glycolytic nodes (e.g., PKM2, ENO1, LDHA)—in line with the redox thiol switch that links sulfur flux to energy metabolism. Sensor cysteines on stress- and inflammation-control hubs such as Keap1 (Cys151/273/288), NF-κB components (RelA/p65 Cys38; IKKβ Cys179) and STAT3 (Cys259) also recur in these datasets and have been validated in targeted kinetic and structural work, supporting a model in which fast, reversible persulfidation tunes pathway gain without forfeiting future responsiveness [16,47,48].

Beyond signaling and glycolysis, recurrent categories include mitochondrial and thiol-redox machinery (Trx/Grx systems), cytoprotective transcriptional scaffolds, and endothelial/immune effectors—mirroring targeted evidence for persulfidation of eNOS, p47^phox^ and caspase-1. Collectively, these surveys delineate a set of persulfidation “hotspots” that couple RSS tone to antioxidant defense, bioenergetic control and innate immunity, and they provide an empirical scaffold that underpins the mechanistic vignettes discussed throughout the review [49,50,51].

Although it shares with S-nitrosylation (–SNO) and S-glutathionylation (–SSG) the function of “shielding” reactive cysteines, persulfidation differs in thermodynamics and resolution machinery. The S–N bond of nitrosothiols is labile to light and metals and is usually broken down via GSNOR, whereas S–S–H is mostly reduced by TRX/GRX. In addition, H_2_S can exchange sulfur with a nitrosothiol, release ^•^NO, and convert –SNO to –SSH, shifting the signal to NRF2 pathways and attenuating nitrosotoxicity [52,53,54].

S-glutathionylation, on the other hand, acts as a “buffer” against peaks of oxidizing species, because glutathione forms a mixed disulfide of +305 Da that transiently blocks reactive cysteine; however, that bulky adduct decreases the nucleophilia of the residue and can inhibit key enzymes such as GAPDH during oxidative stress [55,56]. When the cell needs to reactivate the catalytic function or expand the redox range, the same site can exchange glutathione for a sulfur sulfane atom (–SSH): this persulfidation preserves antioxidant protection, but being smaller and more acidic returns to the residue a larger nucleophilia and a wider redox window, properties that make it the preferred modification to regulate enzymes sensitive to energy status and glycolytic flow [57,58].

Overall, persulfidation emerges as a reversible, chemically versatile, and enzymatically regulated redox switch, supported by detection tools capable of placing it at the center of sulfur-based signaling and opening up new therapeutic opportunities.

### 3.1. Reproducibility and Standardization in RSS Analytics

Across laboratories, reproducibility in RSS measurements is chiefly limited by (i) speciation drift during sampling/derivatization (free H_2_S―persulfides/polysulfides), (ii) method heterogeneity in persulfidation workflows (blocking reagents, click chemistry, and enrichment settings), and (iii) probe/sensor cross-reactivity and passivation that degrade sensitivity over time. While improved tag-switch chemistries and quantitative click-proteomics have expanded coverage, they still under-quantify longer sulfur chains and lack second-to-minute temporal resolution; fluorescent “sulfane–sulfur” probes such as SSP4 remain valuable but require rigorous calibration and selectivity controls. At the clinical interface, bedside amperometric sensors for free H_2_S/polysulfides show coefficients of variation >20% and interference from other acid gases, underscoring the need for harmonized calibration and reporting. Together, these technological limits explain divergent effect sizes and target lists across laboratories and hinder meta-analysis. The following assays and standardizations are most urgently needed:

○ Minimal sample-handling SOPs that stabilize RSS speciation: immediate cold quench, metal chelation and standardized alkylation/blocking prior to lysis to prevent artifactual interconversion (free H_2_S vs. low-MW persulfides/polysulfides vs. protein-SSH); report timing, temperature and pH at derivatization.
○ Community “reference protocol” for the improved tag-switch (reagent identity/concentration, reaction times, click handles, and positive/negative controls), with site-level quantification via isotopically labeled iodoacetamide–alkyne probes to enable inter-lab comparability of occupancy changes.
○ Benchmarking panels for SSP4 and related probes: define dynamic range, response factors for persulfides vs. polysulfides and mandatory orthogonal validation (e.g., parallel tag-switch or MS) to mitigate false positives; include reference datasets and spike-in materials (e.g., GSSH).
○ Reporting standards for speciation and units: always distinguish free H_2_S, acid-labile sulfide, bound/persulfidic pools and protein-SSH; specify tissue compartment, subcellular fraction and donor release kinetics when applicable.
○ Calibration/QA for real-time sensors: two-point calibration before/after runs, interference testing against SO_2_/NO_2_ and other acid gases, and recommended redundant sensing (dual cartridges) in exposure studies, until interferences and passivation are formally resolved

By codifying these assays and minimum-reporting items at the method level and aligning them with clinical monitoring constraints (Section 9.3), the field can reduce inter-lab variance, improve effect-size comparability and accelerate translation from bench to bedside.

## 4. Crosstalk RSS-ROS/RNS. Synergies and Antagonisms in Mitochondria, NADPH-Oxidase and ^•^NO-Synthases

**Figure 6:**
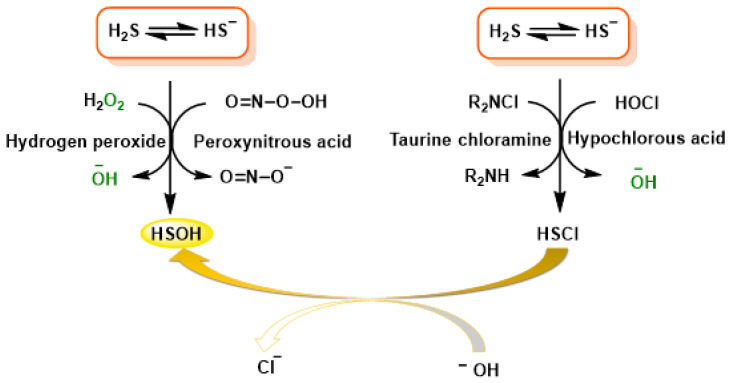
Oxidation of H_2_S by H_2_O_2_, ONOOH and HOCl and production of HSOH.

H_2_S reacts with oxidants such as H_2_O_2_, peroxonitrous acid, hypochlorous acid and chloramines to transform into sulfenic acid (HSOH) which is an unstable intermediate (Figure 6).

The primary outcome of the chemical reaction between H_2_S and hydrogen peroxide (H_2_O_2_) is the formation of hydroxylthiol (HSOH). The final product consists largely of polysulfides, elemental sulfur and, in the case of excess oxidant, sulfate, and it depends on the initial ratios of hydrogen peroxide and H_2_S. The direct reaction between peroxynitrous acid and HS^—^ involves a nucleophilic substitution of HS^−^, leading to the formation of HSOH and NO_2_^−^ as starting products. In the presence of an excess amount of H_2_S, HSOH further reacts with a second HS^−^, leading to the formation of HSS^−^/HSSH and other compounds.

Hypochlorous acid and HS^−^ produces HSCl, subjected to rapid hydrolysis to HSOH. Chloramines, particularly RHNCl and R_2_NCl, exhibit lower reactivity but higher selectivity as oxidants compared to hypochlorous acid, as indicated in Table 3.

Modern redox signaling is no longer conceived as independent circuits of oxygen, nitrogen or sulfur: the three networks share mediators, regulate each other and overlap on key metabolic targets. The most recent literature describes this network as a “dynamic triangle” in which RSS can attenuate, divert or enhance the ROS and RNS pathways depending on the concentration, subcellular location and energy state of the cell [62].

In the mitochondrial compartment, physiological levels of H_2_S enter the respiratory chain through the QRS, feed the ubiquinone and generate a slight decoupling that reduces electron leakage and, therefore, the production of superoxide in complex III. When endogenous H_2_S synthesis falls—for example, after silencing CSE—the mitochondrial ROS fraction increases, while the overexpression of the enzyme dampens this oxidative excess [25]. The scenario is reversed if the sulfide accumulates above the micromolar range: it then reversibly inhibits complex IV, collapses the membrane potential, and triggers secondary radicals, a phenomenon described in models of Down syndrome and acute toxicity [63]. In addition, relatively mild ROS, such as O_2_ or H_2_O_2_, oxidize HS^−^ to polysulfides, creating a deposit of “sulfur sulfan” that can be returned to H_2_S based on redox demand [64]. H_2_S itself also neutralizes peroxynitrite, preventing tyrosine nitration and preserving sensitive mitochondrial functions [65].

On the cell surface and phagosomes, the NOX family constitutes another dialogue interface. The persulfurization of the organizer subunit p47^phox^—or its “prevulcanization”, according to the original nomenclature—prevents its phosphorylation and the complete assembly of the complex, so that H_2_S attenuates the generation of superoxide in the endothelium, kidney and heart [66,67]. This effect explains, in part, the vasculoprotective action of the gas transmitter against hypertension and atherogenesis. However, crosstalk is not unidirectional: in inflammatory cells, sulfur can enhance respiratory explosion—or, indirectly, do so through metabolites such as sulfite—to strengthen the defense against pathogens, underlining its biphasic nature dependent on cell type [7].

The dialogue with the RNS is particularly intricate. In endothelium, H_2_S activates eNOS by Ser1177 phosphorylation and Cys433 persulfidation, increasing ^•^NO flux and enhancing guanylate cyclase-dependent vasodilation [68,69]. Conversely, ^•^NO modulates CBS and CSE activity through S-nitrosylation, thereby modulating sulfide biosynthesis. The two gases can also react chemically to generate mixed intermediates—HSNO and SSNO^−^—that act as stable “relays”, capable of releasing either ^•^NO, H_2_S or polysulfides depending on the redox environment; These intermediates explain synergy phenomena in smooth muscle relaxation and angiogenesis [52,70].

Under physiological conditions, sulfide modulates the production of ROS and RNS, neutralizes them when they exceed toxic thresholds and, at the same time, uses their derivatives (polysulfides and persulfides) to reprogram bioenergetic and transcriptional pathways. When the concentration of H_2_S shifts—due to inflammation, hypoxia, or mutations that inactivate SQR—the balance is disrupted and the signal can shift toward pro-oxidation or nitrosotoxicity. Identifying the “windows” in which the RSS axis behaves as an antioxidant, pro-oxidant or co-messenger is essential to therapeutically exploit its crosstalk with ROS and RNS in cardiovascular, metabolic and inflammatory pathologies [71,72,73,74].

## 5. Physiological Role of RSS

**Figure 7:**
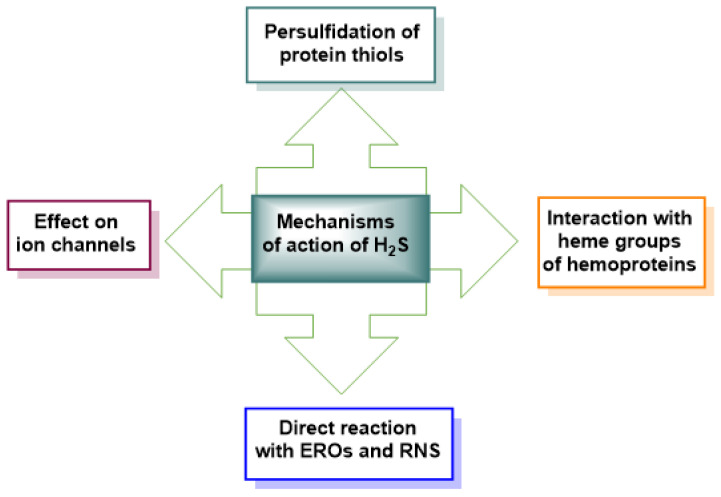
H_2_S signaling mechanisms.

The mechanisms through which H_2_S exerts its effects are not yet fully understood. However, there is sufficient consensus regarding four mechanisms of action or molecular targets [75,76] (Figure 7).

The endogenous production of H_2_S and its persulfidic derivatives is far from being a metabolic “residue”; it constitutes a transverse regulatory axis that synchronizes cellular energy status with vascular function, immune surveillance and neuronal plasticity. In the heart and skeletal muscle, physiological concentrations of H_2_S stimulate the AMPK-PGC-1α pathway and trigger mitochondrial biogenesis programs, thus ensuring optimal ATP supply with lower ROS generation [77]. At the vascular level, sulfide enhances the activity of eNOS and preserves the bioavailability of ^•^NO, forming a synergistic vasodilation circuit that maintains tissue tone and perfusion [78]. In the innate immune system, RSS persulfate caspase-1 and other components of the NLRP3 inflammasome, which attenuates the release of IL-1β and reduces the risk of uncontrolled inflammation [79]. Finally, in the brain, sulfur acts as a neuromodulator and trophic factor: its donors raise BDNF levels and promote survival and synaptic remodeling, crucial effects for learning and resilience in the face of stress [80]. These observations confirm that RSS function as true physiological integrators, capable of linking cellular bioenergetics with vascular, immunological and neuronal homeostasis.

In the mitochondrial matrix, the HS^−^ anion enters the sulfur oxidative pathway and is taken up by the SQR. This flavoprotein directly reduces ubiquinone and transfers the electrons to the I–III set, thereby contributing to the proton-motor gradient (“Q-bypass”) with a lower ROS production than that which accompanies the flow of NADH or FADH_2_ [63]. When the supply of H_2_S is maintained in the nano-micromolar range, the signal is extended to the cytosol: the gas stimulates the AMPK energy kinase, which phosphorylates PGC-1α and sets in motion a mitochondrial biogenesis and oxidative recycling program (Sirt1-dependent) in cardiomyocytes and skeletal muscle; the phenomenon has been proven both with oral donors of H_2_S (SG-1002) and with the overexpression of CSE/CBS [77]. In parallel, the action of SQR keeps electron leakage under control; CDOR-deficient mice or fibroblasts show increased ROS and respiratory dysfunction, underscoring the “antioxidant by-diversion” nature of the circuit [30].

In the cytosolic compartment, excess HS^−^ or polysulfides (Sn^2−^) persulfate the catalytic cysteine of GAPDH (Cys156), inhibiting its activity and causing the accumulation of glyceraldehyde-3-phosphate. As a consequence, glucose is redirected to the oxidative branch of the pentose pathway, increasing the production of NADPH and strengthening antioxidant defenses [36]. This metabolic “valve cut”, triggered by sulfur sulfan, complements the reduction of ROS in the respiratory chain and exemplifies how RSS coordinates energy balance with redox protection.

In the endothelium, H2S amplifies eNOS activity by two complementary pathways: (i) activates the Akt kinase and promotes phosphorylation of the Ser1177 residue, which doubles ^•^NO production after 30 min of exposure to 25–150 μM H_2_S [81]; and (ii) persulfates the Cys4333 residue, stabilizing the dimeric form and prolonging the half-life of the enzyme [82]. The result is a sustained increase in ^•^NO flow which translates into higher levels of cGMP and smooth muscle relaxation.

The interdependence between the two gas transmitters has been demonstrated in gain-loss of function models: H_2_S-induced vasodilation is drastically attenuated in aortic rings of eNOS^−^/^−^ mice, and, conversely, CSE silencing reduces vessel response to AED/NO or acetylcholine; the addition of permeable cGMP restores relaxation, underlining that the point of convergence is the ^•^NO–sGC–cGMP pathway [83]. Similarly, in in vivo angiogenesis assays (Matrigel plug, dermal burns) H_2_S loses its pro-vascular effect if eNOS is inhibited, while disruption of sulfide synthesis cancels out neovascularization stimulated by VEGF or acetylcholine [83]. These findings confirm that vasorelaxation and angiogenesis depend on a cooperative H_2_S/NO circuit: the sulfide persulfide and phosphorylated eNOS, elevates ^•^NO and, in turn, ^•^NO maintains the expression and activity of trans-sulfuration enzymes, ensuring a continuous flow of both mediators to preserve vascular tone and tissue perfusion.

Macrophages and the intestinal epithelium use the reactive sulfur axis as a “molecular brake” on the inflammasome. In BMDM murine macrophages and in THP-1 human monocytes, slow H_2_S donors such as GYY4137 or thiosulfate (25–100 μM, releasing <1 μM of free sulfide) suppress the secretion of IL-1β and IL-18 induced by urate or nigericin crystals. The mechanism combines (i) fall of mitochondrial ROS and ASC assembly with the consequent loss of caspase-1 activity and (ii) persulfidation of the Cys163 catalytic residue of caspase-1, which blocks its proteolytic capacity [79].

The importance of endogenous sulfide is genetically confirmed: macrophages from CSE^−^/^−^ mice -or treated with the PAG inhibitor- show a release of IL-1β up to twice as much and a parallel increase in the activation of the NLRP3 complex; this phenotype is reversed by replenishing H_2_S or by introducing polysulfides, underlining the regulatory function of S-sulfydration [49].

In the gut, CBS is the main source of sulfur. Models of colitis in CBS^+^/^−^ mice reveal that the chronic drop in H_2_S promotes inflammatory hyperactivation, deteriorates the epithelial barrier and increases COX-2 expression. The exogenous contribution of GYY4137 restores neighborhood integrity by inhibiting the inflammasome and increasing the sulfydration of the HuR protein that controls the stability of the COX-2 mRNA [84].

Overall, sub-micromolar concentrations of H_2_S or its derived polysulfides are sufficient to keep the NLRP3 inflammasome silenced by direct caspase-1 persulfidation and limitation of oxidative stress, while sustained loss of the CBS/CSE axis enhances sterile and bacterial inflammation. These data reinforce the idea that adjusting the endogenous sulfide “window” may be a therapeutic strategy to control NLRP3-dependent systemic and gastrointestinal inflammatory diseases [85].

In the central nervous system, H_2_S behaves both as a fast neuromodulator—it facilitates long-term potentiation by enhancing NMDA current—and as a slow-acting trophic factor. In several models of chronic unpredictable stress (CUMS), continued administration of slow H_2_S donors (NaHS 0.03–0.1 mmol kg^−1^ day^−1^ or GYY4137 25–100 μM) prevents loss of dendritic spines in CA1 and restores spatial memory in Morris’s labyrinth by inducing BDNF expression and phosphorylation of its TrkB receptor in the hippocampus [86]. Activation of the BDNF/TrkB axis, in turn, triggers the PI3K-Akt-CREB pathway and reinforces survival and neuritic growth; pharmacological inhibition of endogenous sulfur synthesis with propargill-glycine (PAG) or CSE/CBS deletion reverses these benefits and reproduces a depressive phenotype with decreased BDNF and increased microgliosis [87,88]. Beyond stress, in models of Parkinson’s and traumatic brain injury, H_2_S donors preserve dopaminergic viability and accelerate neurogenesis, an effect that is attenuated after blocking TrkB or silencing BDNF, confirming that sulfhydric signaling converges on this trophic pathway [89,90].

Together, RSS links cellular metabolism with vascular, immune and neuronal signaling. That multifunctionality, based on the reversible chemistry of sulfur sulfan, explains why small variations in H_2_S production or catabolism can tip the physiological balance toward metabolic protection or, if balance is disturbed, toward cardiovascular dysfunction, chronic inflammation or cognitive decline.

## 6. RSS in Cardiovascular Disease, Aging, Sarcopenia, Neurodegeneration, Cancer and Long COVID

We classify the evidence cited in this review as preclinical (cell/animal), human observational or interventional clinical (phase I–III). We apply these descriptors consistently in this Section and summarize key uncertainties for each indication. This clarification complements our synthesis of mechanistic RSS biology and therapeutics and aligns claims with the underlying level of validation. Across indications, two cross-cutting gaps remain: (i) analytics, i.e., standardized, real-time quantification of persulfide/polysulfide fluxes in humans; and (ii) therapeutic windows, given the narrow range between protective signaling and cytotoxic inhibition of respiration—both central to trial design and interpretation. We therefore qualify conclusions accordingly and highlight where claims are hypothesis-generating rather than practice-changing.

**Figure 8:**
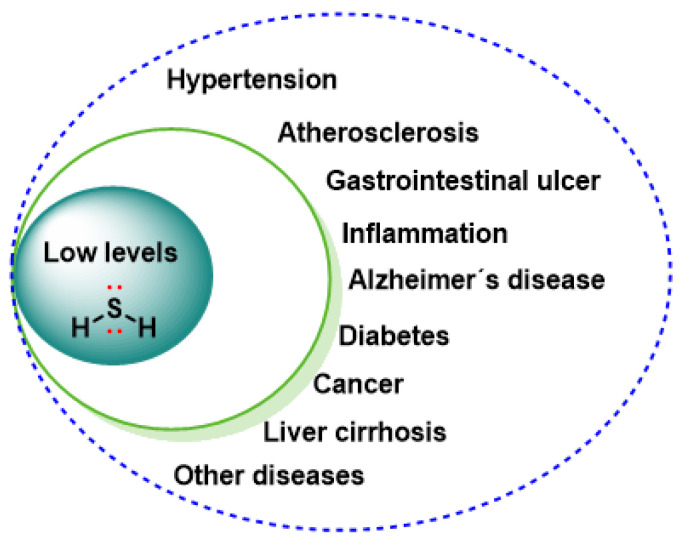
Examples of diseases related to low levels of H_2_S.

Figure 8 provides a comprehensive representation of the biological mechanisms in human physiology that are controlled by endogenous H_2_S or show a response to pharmacological intervention with H_2_S or its derivatives.

An imbalance in the biosynthesis, catabolism, or signaling of RSS is involved in the genesis and progression of multiple chronic diseases. Key findings in five high-impact clinical settings are summarized below:

- **Cardiovascular disease**. Evidence: robust preclinical cardioprotection (ischemia–reperfusion, remodeling) and early interventional clinical signals with oral slow-release donors (e.g., SG1002) showing biomarker shifts (↑H_2_S/NO surrogates, ↓BNP) and acceptable safety. Uncertainties/controversies: paucity of hard outcomes, optimal dose and kinetics (salt vs. slow donors vs. mitochondria-targeted), responder phenotypes, and interaction with standard HF therapies. The bioavailability of H_2_S is reduced proportionally to the severity of heart failure: a study of 124 patients with varying degrees of congestion showed total sulfur concentrations of ≈5.3 [2.2–8.0] μM versus 8.5 [6.0–14.0] μM in healthy controls, and the decline was accompanied by reduced endogenous capacity to produce H_2_S and depressed CSE activity [91]. Preclinical models confirm the causal relationship: mice with cardiac overexpression of CSE or treated with inorganic donors (NaHS 3 mg kg^−1^ day^−1^) have smaller infarct size, better diastolic relaxation and less remodeling after ischemia–reperfusion, while genetic CSE deficiency aggravates functional impairment [92,93]. Clinical translation has begun with the oral prodrug SG1002: in a phase I/II trial in patients with NYHA II–III, step doses of 200–800 mg bid steadily increased plasma H_2_S, elevated nitrite (indicator of ^•^NO), and attenuated the increase in BNP without causing symptomatic hypotension or electrocardiographic abnormalities. endorsing its safety and potential hemodynamic benefit [18]. Taken together, these data support that strengthening the CSE/H_2_S axis—either through gene therapy or drug donors—offers a promising strategy for limiting ischemic injury, improving diastolic function and slowing the progression of heart failure.
- **Aging and sarcopenia**. Evidence: preclinical data indicate that restoring H_2_S supports autophagy, mitochondrial biogenesis, and myofiber integrity; limited ex vivo human myotube findings. Uncertainties: translatability to older adults, long-term safety and net effects on proteostasis under comorbidities.
  During aging, the expression of CSE—the main enzyme that generates H_2_S in muscle—decreases steadily, and its loss aggravates age-related atrophy: in old mice, CSE deficiency triggers loss of lean mass and slows regeneration after cardiotoxin, while NaHS supplementation restores myogenic genes and accelerates fiber repair [94]. In parallel, reduced sulfur synthesis raises muscle ROS and contributes to the blockade of basal autophagy; recent studies have shown that replenishing the “H_2_S axis” with slow donors such as GYY4137—or, genetically, overexpressing CSE/CBS—reactivates the AMPK pathway → PGC-1α/ULK1, stimulates autophagy and mitochondrial biogenesis, reduces proteolysis and preserves fiber diameter, effects that translate into a significant prolongation of the half-life of senescent mouse models [95,96]. In humans, the relevance is parallel: myotubes derived from vaso-lateral biopsies exposed to sulfide or polysulfide donors show lower expression of the ubiquitin MuRF1/atrogin-1 ligases and greater induction of mitochondrial genes dependent on PGC-1α, indicating that the reactive sulfur signal retains the ability to slow down proteolysis and improve energy quality also in human tissue in vitro [97]. These findings place RSS as a promising target to counteract primary sarcopenia, either by boosting endogenous production (cysteine-rich nutraceuticals, CSE/CBS activators) or by administering controlled release clinical donors of H_2_S.
- **Neurodegeneration**. Evidence: predominantly preclinical (Parkinson’s/Alzheimer’s models: anti-inflammatory, anti-aggregative, BDNF/TrkB support). Uncertainties: brain delivery and chronic dosing of donors, cell-type specificity (neurons vs. microglia), and disease-stage heterogeneity.
  In the brain, physiological levels of H_2_S < 1 μM insulate the neuron from the double excitotoxic-oxidative stress: the gas neutralizes the excess Ca^2+^ that follows the overactivation of NMDA receptors and, in parallel, prevents α-synuclein from undergoing nitration and aggregating. In the MPTP model of Parkinson’s, the slow donor GYY4137 (50 mg kg^−1^ i.p.) preserves tyrosine hydroxylase activity, reduces α-synuclein nitration, and maintains the density of dopaminergic neurons in the substantia nigra; Under the same conditions, motor coordination improves in the Rotarod test [98]. Complementary studies with 6-OHDA show that chronic administration of NaHS (30–100 μmol kg^−1^) or polysulfides increases the survival of nigro-striatal neurons through the opening of K_ATP channels and the suppression of microgliosis [99].

The sulfur signal extends to the gut–brain axis: sulfate-reducing bacteria supply H_2_S that modulates epithelial permeability, vagal tone and cytokine production; recent reviews link this microbial pathway with the progression of both Parkinson’s and Alzheimer’s, highlighting that dysbiosis with loss of sulfur producers accelerates neuroinflammation and the deposition of misfolded proteins [100]. In Alzheimer’s models, sulfydration of GSK-3β decreases Tau hyperphosphorylation and administration of NaHS attenuates β-amyloid-induced neuroinflammation in BV-2 microglia, preserving cell viability [101].

Taken together, the preclinical data support that strengthening the nanomolar “window” of RSS—through slow donors, foods rich in sulfur compounds or modulators of the microbiota—can slow dopaminergic loss, dampen neuroinflammation and delay the amyloid-Tau cascade, placing reactive sulfur as a promising target against neurodegenerative disorders.

- **Cancer**. Evidence level: preclinical and dominant signals: CBS/CSE/3-MST overexpression, tumor “sulfur addiction”, and xenograft sensitivity to enzyme inhibition (e.g., AOAA) or high-load donors (e.g., GYY4137). Key uncertainties/controversies: bell-shaped (biphasic) dose–response; tumor-type heterogeneity; effects on the immune microenvironment; optimal dosing kinetics and delivery; predictive biomarkers for patient selection.

Tumor cells systematically reprogram their sulfur metabolism to sustain bioenergetics, angiogenesis and drug resistance. In the colon, malignant epithelium selectively overexpresses cystathionine-β-synthase (CBS); the H_2_S it produces maintains oxidative phosphorylation and promotes cell migration, while silencing CBS or inhibiting it with AOAA reduces proliferation in vitro and slows the growth of murine xenografts [102]. In the ovary, both CBS and CSE are elevated and their abundance is associated with a worse prognosis, greater immune infiltration, and resistance to platinum; CSE overexpression increases the formation of polysulfides that neutralize cisplatin-induced oxidative stress, while blocking the enzyme reverses chemoresistance [103,104]. Lung tumors and colorectal carcinomas that develop resistance to 5-fluorouracil further show induction of 3-mercaptopiruvate sulfurtransferase (3-MST), suggesting metabolic “back-up” when CBS or CSE are limiting [105,106].

Functionally, the action of H_2_S follows a bell curve: low-moderate endogenous concentrations (<100 μM in tissue) favor tumor survival, while supra-micromolar doses—administered with “high-load” or rapid-release donors—collapse mitochondrial potential, elevate ROS and trigger apoptosis or selective necroptosis of the cancer cell [107,108,109]. This duality explains why two opposing but complementary strategies are being explored: (i) inhibiting H_2_S biosynthesis with CBS/CSE/3-MST blockers to deprive the tumor of its bioenergetic advantage and (ii) overloading the tumor with high-dose donors (e.g., rapid polysulfide releasers) that push intratumoral concentration into the cytotoxic range. Studies in colon and ovarian xenografts show that both approaches—AOAA or GYY4137 at 200 mg kg^−1^—reduce tumor volume without affecting fibroblasts or healthy tissues, supporting the therapeutic viability of “targeted sulfur” [110].

Together, CBS/CSE/3-MST enzyme overexpression confers a metabolic dependence on sulfur in multiple tumors; Taking advantage of that addiction—either by blocking its source or by saturating the machinery with lethal doses of sulfur—emerges as a promising antitumor strategy.

At present there are no validated interventional clinical data in oncology; platform viability is inferred from robust preclinical signals and from the demonstrated clinical tolerability of H_2_S donors in non-oncology indications (see Section 9). Crucially, the biphasic nature of H_2_S biology—where low–moderate exposure can support tumor fitness while higher exposure is cytotoxic—imposes a central design constraint for first-in-human studies. Accordingly, early trials should prospectively stratify patients by CBS/CSE/3-MST expression (prognostic in ovarian cancer and likely predictive of sulfur-targeted strategies), choose release kinetics (slow vs. rapid) to match the intended mechanism (signaling modulation vs. cytotoxicity), and incorporate orthogonal speciation analytics to verify on-target sulfur exposure in tumor versus stroma. These guardrails will maximize interpretability and safety as the field advances toward definitive efficacy testing.

- **Long COVID**. Evidence level: human observational + interventional pilot; dominant signals: sustained thiol (R-SH) depletion correlating with dyspnea/fatigue, and improved FEV_1_, mMRC, IL-6/CRP after double-blind sulfurous inhalations (~30 ppm). Key uncertainties/controversies: durability of benefit; generalizability beyond respiratory endpoints; replication in larger, multi-center cohorts; head-to-head vs. classical H_2_S donors; standardized analytics for target engagement.

A longitudinal follow-up of 135 patients with mild infection showed that, at 3, 6, and 12 months, serum R-SH levels remained markedly depressed compared to healthy controls; the magnitude of this depletion was correlated with the intensity of fatigue and dyspnea, confirming that sustained oxidative stress and loss of the thiolic pool are biochemical features of post-COVID syndrome [111]. At the mechanistic level, cell studies and studies in animal models showed that moderate elevations of H_2_S—obtained either by gene induction of CBS/CTH or with slow donors—restore mitochondrial coupling, normalize NADH/NAD^+^ balance, and reduce ROS production, resulting in a two-order drop in SARS-CoV-2 [112] viral load. The translational potential of this pathway is beginning to be validated: a double-blind pilot trial in 30 patients with long COVID showed that 12 sessions of sulfurous thermal water inhalations (≈30 ppm H_2_S) improved FEV_1_, reduced the mMRC dyspnea index, and normalized IL-6 and C-reactive protein compared to distilled water inhalations [113]. The data suggest that restoring the sub-micromolar sulfur “window”—either with pharmacological donors or balneotherapeutic interventions—could mitigate persistent oxidative stress and accelerate functional recovery in long COVID. Early signals justify phase II studies powered for functional and quality-of-life endpoints, with standardized speciation readouts (free H_2_S, sulfane sulfur, protein-SSH) to confirm target engagement, and comparison of inhalation vs. systemic donors. Given ongoing uncertainties, claims remain hypothesis-generating pending larger trials with longer follow-up.

Taken together, this evidence places RSS at the center of modern pathophysiology: its deficiency or overproduction can tip the balance towards cardiovascular injury, muscle and neuronal deterioration, tumorigenesis or viral sequelae. Understanding the thresholds that convert reactive sulfur from protectant to pathogen is key to designing next-generation dietary and pharmacological interventions.

## 7. Exogenous Modulation. H_2_S Pharmacological Donors, Smart Releasers, Dietary Compounds and Sulfur-Producing Microbiota

There is a growing interest in selectively modulating and elevating RSS when endogenous production is insufficient—or, at the opposite extreme, to saturate tumor cells or sulfur-sensitive pathogens in a controlled manner. These approaches include first- and second-generation pharmacological donors, “smart” platforms with regulated release, sulfur nutrients and the manipulation of the H_2_S-producing intestinal microbiota:

- **Classic and slow-release drug donors**. The inorganic salts sodium-hydrosulfide (NaHS) and sodium-sulfide (Na_2_S) continue to be the most widely used tools in vitro because they dissociate almost instantaneously and reach millimolar peaks of H_2_S; however, this brief and difficult to dose “burst” moves away from physiology and limits its clinical translation [114]. To overcome this obstacle, oral prodrugs and extended-release donors have been developed. SG1002 polysulfide—formulated in 200–800 mg capsules twice daily—was safe in both healthy volunteers and patients with heart failure (NYHA II-III); the 21-day treatment stably raised plasma levels of H_2_S and nitrite, and attenuated the rise in BNP and systolic blood pressure, results that supported its move to larger phase II studies [18]. Preclinically, thiobenzide GYY4137 releases less than 20 μM of H_2_S over several hours-days; this profile better mimics endogenous synthesis and has been linked to post-ischemic cardioprotection, antineoplastic action and anti-inflammatory effects with a much lower toxicity than that of fast salts [115,116]. With this evidence, slow-release donors are emerging as the most promising platform to restore the H_2_S pool in chronic pathologies without exceeding the cytotoxic threshold.
- **“Smart” and organelle-directed donors**. The second wave of compounds incorporates chemical “triggers” that deliver H_2_S only in specific micro-environments, avoiding the toxic spikes of fast salts. A first example is the aromatic gem-dithiol, which remain inert until they react with cellular thiols (cysteine, GSH); In doing so, they release the gas in a stoichiometric and controllable way both in physiological buffer and inside the cell [117].

To direct the sulfide to the mitochondria, the lipophilic tripeptide AP39 was designed, to which the triphenylphosphonium cation (TPP^+^) is grafted. The TPP^+^ vector drags the molecule into the mitochondrial matrix, where AP39 protects mitochondrial DNA, enhances endothelial bioenergetics, and—in a recent study—attenuates doxorubicin cardiotoxicity by modulating the pathway [118].

Other platforms combine H_2_S with complementary gas transmitters: NOSH-aspirin hybrids (and “NOSH-NSAID” analogues) bind a nitrate (NO) and a sulfur-releasing motif in a single drug, showing greater anti-inflammatory and cytotoxic potency against tumor cells than their mono-releasing precursors [119,120,121]. In parallel, photo chromophores have been described that release H_2_S (or H_2_S_2_) under visible light or NIR, allowing such fine spatiotemporal control that it is even possible to monitor the release in real time by changing fluorescence [122,123,124].

Together, these “smart” donors—activatable by thiols, light, or subcellular localization—significantly expand the pharmacological arsenal and offer the possibility of calibrating the sulfur dose right at the therapeutic target, a key requirement to exploit its potential without exceeding the cytotoxic threshold.

- **Sulfur dietary compounds**. Sulfur-rich foods illustrate how nutrition can modulate the RSS pool without resorting to drugs. In garlic, lipophilic polysulfides—notably diallyl trisulfide (DATS)—are stable donors that release H_2_S in situ: in a mouse model of myocardial ischemia–reperfusion, DATS elevated plasma sulfide, reduced infarct size, and preserved ventricular function [125]; in addition, in rats with metabolic syndrome it improved lipid profile and attenuated cardiac dysfunction, effects linked to the sustained increase in H_2_S and the fall of pro-oxidant species [126].

**Figure 9:**
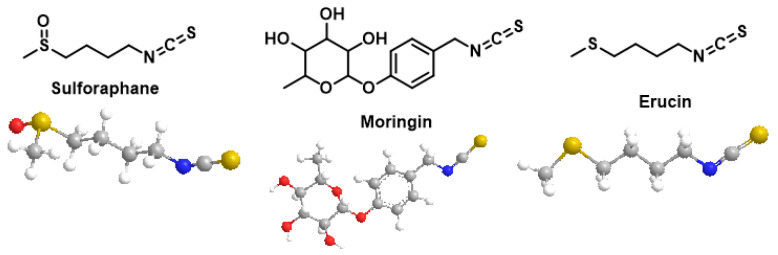
Molecular structures of sulforaphane, erucine and moringin isothiocyanates.

Among cruciferous vegetables, sulforaphane, erucine and moringin isothiocyanates, Figure 9, release sulfide more slowly than garlic polysulfides. Sulforaphane achieves micromolar concentrations of H_2_S in liver homogenates and its antiproliferative action on PC-3 prostate cells depends, at least in part, on this sulfur release [127,128]. Erucine, on the other hand, acts as a vasodilator and hypotensive: the persulfidation of Kv7 channels shifts the activation threshold and promotes the relaxation of vascular smooth muscle [129,130,131].

The bioavailability of these phyto-donors depends on the culinary processing and the microbiota. Heat deactivates the plant myrosinase responsible for hydrolyzing glucosinolates; When the enzyme is lost (prolonged cooking), the conversion to isothiocyanates is relegated to intestinal bacteria, which may or may not complete the reaction depending on their composition [132,133,134]. Thus, both the way of cooking (short steam rather than boiled) and the abundance of myrosinase-positive microbes condition the actual dose of sulfur that reaches the circulation.

Together, garlic polysulfides and cruciferous isothiocyanates provide a dietary pathway to strengthen the H_2_S signal. The modulation of the microbiota and culinary techniques offers additional scope to optimize their clinical effectiveness.

- **Sulfur-producing microbiota**. In the colon, sulfate-reducing bacteria (SRB), led by *Desulfovibrio* spp., metabolize sulfates and sulfur amino acids to release H_2_S. When production is maintained in the nanomolar range, the gas supports the integrity of the intestinal barrier—for example, the CBS-H_2_S persulfidal pathway to the HuR protein reduces COX-2 expression and protects the epithelium against lipopolysaccharide [84]. However, the overpopulation of SRB typical of dysbiosis in ulcerative colitis or metabolic syndrome raises sulfur to millimolar concentrations that damage colonocytes, increase permeability, and fuel neuroinflammation at a distance [135,136].

The microbial production of H_2_S is modulable. Diets rich in soluble fiber and polyphenols shift the ecosystem toward beneficial fermenters and reduce sulfur generation, while excess protein increases the SRB load; Introducing fiber reverses this effect and normalizes the luminal redox balance [137]. Beyond diet, probiotic strategies are gaining ground: competing strains that consume SRB substrates decrease sulfate pressure, and *E. coli* prototypes designed to express sulfide–quinone–oxidoreductase in situ titran the gas, keeping it in the therapeutic “window” and mitigating inflammation in both human epithelial cultures and microphysiological models of the colon [138]. These approaches open the door to “taming” the sulfhydryl signature of the microbiota—either by reducing toxic peaks, or by reinforcing the protective tone—as a nutritional supplement or as a precision medicine tool.

- **Operational synthesis**. The current therapeutic repertoire allows several layers of intervention to be superimposed on the H_2_S axis:
  (1) Endogenous production can be enhanced with a diet rich in alliums (garlic, leek) and cruciferous vegetables: the lipophilic polysulfides of garlic and the isothiocyanates of sulforaphane release H_2_S in situ and raise the RSS “pool” in peripheral tissues without generating toxic peaks, as has been proven both in cell assays and in studies on myocardial perfusion and tumor viability [128,139].
  (2) To stabilize circulating levels, slow-release oral donors—for example, thiobenzamide GYY4137—maintain sub-micromolar concentrations of H_2_S for hours and have already demonstrated cardioprotection and fibrosis reduction in hypertensive models, with a toxicity profile lower than that of inorganic salts [140].
  (3) When organelle-cellular specificity is required, “smart” platforms such as AP39 are used, which directs sulfide to the mitochondrial matrix and improves endothelial bioenergetics or neuronal survival in various injury models [141]. (4) Finally, the colonic microbiota can be modulated with fiber, polyphenols, or strains designed to oxidize excess sulfur, so that bacterial production is maintained in the protective nanomolar window without escalating to cytotoxic levels associated with colitis and neuroinflammation [142].

The sequential combination of these approaches—diet to stimulate basal synthesis, slow donors to sustain it, targeted vectors to “fine-tune” the signal where it is most needed, and a “domesticated” microbiota to avoid luminal overload—offers a flexible palette with which to adjust sulfide to the therapeutic range and turn it into a true pharmacological ally without exceeding the cytotoxic threshold.

## 8. Selective Persulfidation and S-Alkylation of Reactive Cysteines of Proteins and Transcription Factors

Sulfhydration of cysteine residues and nitration of tyrosine are H_2_S-induced post-translational modifications induced by H_2_S and RDS, respectively. S-Persulfidation occurs when a thiol group (R–SH) is converted into a persulfide (R–SSH), creating a unique post-translational modification that underlies many of H_2_S’s signaling roles. By attaching an extra sulfur atom to reactive cysteine residues, persulfidation can alter a protein’s structure, activity, stability, or localization. In this way, the dynamic formation (and removal) of persulfides not only regulates enzyme function and signal transduction pathways but also taps into the body’s broader sulfur pool, linking H_2_S signaling to cellular sulfur homeostasis [75].

**Figure 10:**
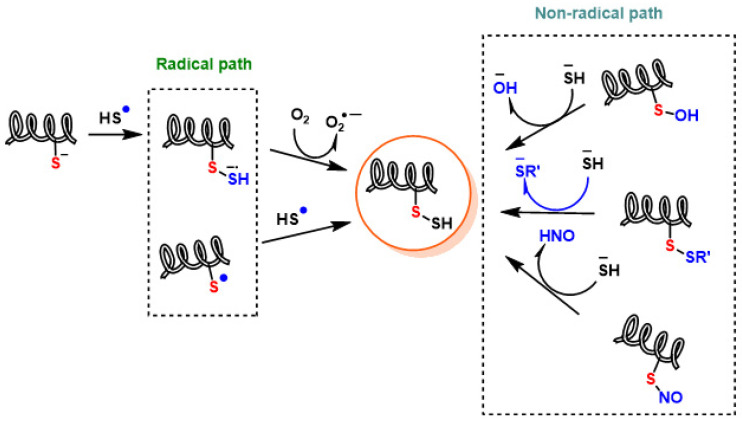
Mechanisms of protein persulfidation.

Persulfides may also arise through radical chemistry, albeit to a minor extent in vivo due to the low concentrations of radical species. In one pathway, the sulfhydryl radical (HS^•−^) can combine with a thiolate anion (RS^−^) to form R–SSH, though this reaction is largely negligible biologically. Alternatively, HS^•−^ can attack a non-radical thiol (R–SH), yielding the radical anion RSSH^•−^. This intermediate then transfers its unpaired electron to molecular oxygen, producing persulfide (R–SSH) alongside the superoxide radical anion (O_2_^•−^) (Figure 10) [41,143].

Electrophilic small-molecule drugs classically silence signaling proteins by forging irreversible C–S Michael adducts with sentinel cysteine residues, a covalent cul-de-sac that removes the thiol from cellular redox circuits and locks the target in an inactive state [144,145]. In stark contrast, HS^−^ and its derived inorganic and low-molecular-weight polysulfides perform a far more agile chemistry: the nucleophilic anion donates one zero-valent sulfur atom to a cysteine thiolate, transiently upgrading it to a persulfide (protein-SSH) [5,44]. The reaction completes on the sub-second time-scale, remains fully reversible thanks to the thioredoxin, glutaredoxin and glutathione systems, yet perturbs the local electrostatics, steric profile and hydrogen-bond network sufficiently to re-route signaling flux [146,147]. Persulfidation therefore behaves as a dynamic rheostat, whereas S-alkylation functions as a one-way switch [6]. To dissect this dichotomy, we focus on three paradigmatic redox sensors—Keap1, NF-κB and STAT3—and analyze how persulfidation of their reactive cysteines modulates pathway activity relative to irreversible alkylation.

### 8.1. Selective Persulfidation and S-Alkylation of Reactive Cysteines in Keap1

**Figure 11:**
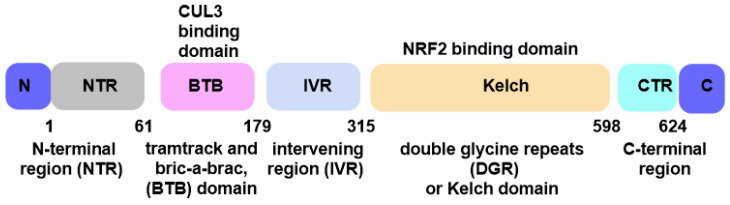
KEAP1 consists of 624 amino acids and has five discrete protein domains: N-terminal region, BTB, IVR, DGR and C-terminal region (CTR).

Kelch-like ECH-associated protein 1 (Keap1) contains twenty-seven cysteine residues and five discrete protein domains: the N-terminal region; the Bric-a-brac/Tramtrack/Broad (BTB) domain (which contains Cys151); the intermediate region domain (IVR), which contains a group of cysteines important for stress sensing, including Cys273 and Cys288; the double glycine repeat (DGR) and C-terminal region (CTR) domains collaborate to form a β-helix structure, which is where Keap1 interacts with NRF2 and forms the Kelch domain. These reactive cysteines under oxidative and electrophilic stress conditions can undergo oxidation or covalent adduct formation, respectively, leading to destabilization of the NRF2/Keap1 complex, and act as redox switches that decide whether Keap1 retains the transcription factor NRF2 on the CUL3/RBX1 ubiquitin–ligase scaffold or releases it to initiate a cytoprotective transcriptional response [148,149], Figure 11.

Under resting conditions these hotspots remain in their highly nucleophilic thiolate state and are therefore primed for persulfidation, a reversible modification that converts a cysteine into a persulfide (–SSH) by accepting a zero-valent sulfur atom from glutathione persulfide, cysteine persulfide or low-molecular-weight polysulfides generated downstream of the cystathionine γ-lyase/cystathionine β-synthase/3-mercaptopyruvate sulfurtransferase axis [37,143].

Persulfidation lengthens the sulfur side chain by roughly 1.8 Å and raises its pKa, introducing a subtle steric and electrostatic disturbance that is sufficient to weaken the Keap1–CUL3 interface but small enough to leave the global fold intact [143,150]. Stopped-flow kinetics and high-resolution mass spectrometry show second-order rate constants in the 10^4^–10^5^ M^−1^ s^−1^ range for Cys151 persulfidation by hydrophilic persulfides, markedly faster than the 10^2^–10^3^ M^−1^ s^−1^ observed for classical α,β-unsaturated electrophiles [151,152]. Cryo-electron microscopy snapshots reveal that the terminal sulfur establishes transient hydrogen bonds with Arg135 and Lys131, promoting a breathing motion that disfavors NRF2 docking [150]. Because the newly added sulfur atom remains nucleophilic, the Cys-SSH adduct is rapidly reduced back to Cys-SH within seconds by thioredoxin, glutaredoxin or the glutathione/GS-persulfide couple, allowing Keap1 to recapture NRF2 as soon as the oxidative burst subsides [16,147]. Importantly, persulfidation also protects the same cysteines from irreversible over-oxidation: sulfenic intermediates formed during H_2_O_2_ exposure are “rescued” to persulfides, preventing their progression to sulfinic or sulfonic states and preserving Keap1’s ability to cycle [151].

NRF2 activation further boosts persulfidation in a feed-forward loop because it induces the transcription of CSE and 3-MST, thereby increasing endogenous persulfide flux and reinforcing its own liberation in a self-limiting pulse [153]. In cellular models, a two-fold rise in persulfide levels appears within thirty seconds of menadione treatment and correlates inversely with global protein sulfinylation, underscoring the kinetic supremacy and protective nature of the –SSH modification.

Irreversible S-alkylation of the same cysteines by α,β-unsaturated carbonyls such as dimethyl fumarate, sulforaphane or endogenous itaconate proceeds at least two orders of magnitude more slowly and locks Keap1 in an inactive conformation until new protein is synthesised, providing a prolonged NRF2 signal but at the cost of thiol depletion and potential off-target toxicity [154,155,156]. This slower, energetically costlier pathway becomes relevant only under sustained electrophilic stress when the persulfide buffer is overwhelmed [157].

**Figure 12:**
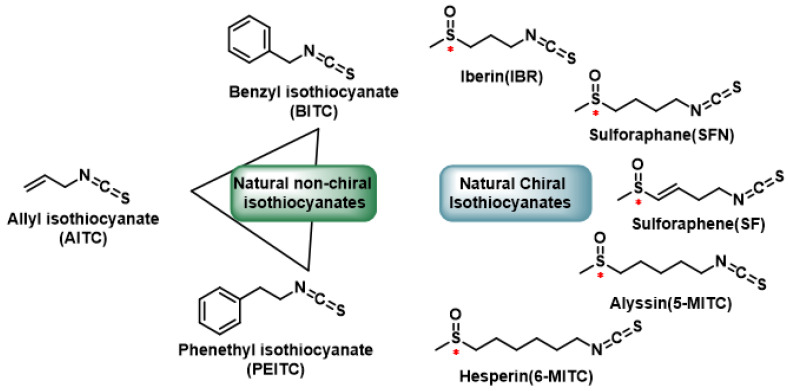
Chemical structure of natural chiral and non-chiral isothiocyanates. Red asterisks indicate the stereogenic sulfur atom (sulfoxide center).

Most activators of the Keap1-NRF2 pathway are classified as electrophiles, which react with individual cysteine residues in Keap1 to form a C-S bond with the thiol group of the cysteine. All isothiocyanates, Figure 12, react with Cys-Keap1 through a conserved mechanism: the electrophilic carbon within the –N=C=S group undergoes a Michael addition with thiol (-SH) groups on specific cysteine residues of Keap1, forming a stable sulfur–carbon bond. This covalent modification disrupts Keap1’s ability to ubiquitinate Nrf2, preventing its proteasomal degradation. Consequently, Nrf2 accumulates in the cytosol, translocates to the nucleus, and binds to antioxidant response elements (AREs) in the genome, inducing the expression of cytoprotective phase II detoxification enzymes such as HO-1 and NQO1 [158,159] (Figure 12).

Thus, persulfidation emerges as the physiologically dominant, rapid-cycling and thiol-protective mechanism for modulating Keap1, whereas S-alkylation represents a blunt, last-resort “fail-safe” that the cell recruits under severe or chronic electrophile load. The convergence of structural, kinetic and in vivo data therefore points to persulfidation as the primary governor of Keap1 status, offering a blueprint for therapeutic interventions that harness transient persulfide donors to mimic endogenous regulation with superior temporal fidelity and safety compared with irreversible electrophilic agents [37,147].

### 8.2. Selective Persulfidation and S-Alkylation of Reactive Cysteines in NF-kB

The canonical NF-κB pathway is governed by a triplet of redox-sensitive cysteines that sit at strategic control points along the signaling axis [160]. At the upstream kinase layer, Cys179 nestles in the activation loop of IKKβ; one tier below [161], the DNA-binding cores of the transcription factors carry Cys38 in RelA/p65 [162] and Cys62 in p50 [163]. All three residues exhibit partial thiolate character at physiological pH and therefore constitute privileged targets for persulfidation [160]. The main characteristics of the persulfidation and s-alkylation of NF-kB are as follows:

- **Persulfidation as the default rheostat**. Persulfidation has emerged as the fast “volume-control” that tunes every tier of the canonical NF-κB pathway. Stopped-flow competition experiments on a well-characterised sulfenic-acid model (Mt AhpE–SOH) give a pH-independent second-order rate constant of 1.4 × 10^3^ M^−1^ s^−1^ for attack by HS^−^, after correction for protonation equilibria. This value sits squarely inside the 1–3 × 10^4^ M^−1^ s^−1^ window quoted for persulfide formation on IKKβ Cys179 [146].

Stopped-flow kinetic analysis revealed that persulfidation of protein cysteines by hydrophilic persulfides occurs with second-order rate constants in the 10^4^–10^5^ M^−1^ s^−1^ range, markedly faster than classical α,β-unsaturated electrophiles. The thioredoxin system acts as a major protein depersulfidase that controls H2S signaling, showing almost 10-fold higher reactivity towards cysteine persulfide than towards cystine [16,39].

NaHS ameliorates TMAO-induced NF-κB activation. The TMAO-induced phosphorylation of p65 NF-κB was reversed by NaHS. In addition, NaHS decreased the expression levels of the pro-inflammatory cytokines IL-1β, IL-6 and TNF-α in TMAO-stimulated macrophages [164,165].

Using an improved tag-switch assay for persulfide detection we show here that protein persulfide levels are controlled by the thioredoxin system. Recombinant thioredoxin showed an almost 10-fold higher reactivity towards cysteine persulfide than towards cystine and readily cleaved protein persulfides as well. Inhibition of the thioredoxin system caused an increase in intracellular persulfides, highlighting thioredoxin as a major protein depersulfidase [16].

Taken together, studies support a model in which sub-micromolar surges of low-molecular-weight persulfides modify the entire NF-κB circuitry within seconds, after which Trx/Grx rapidly return it to the basal state.

**Figure 13:**
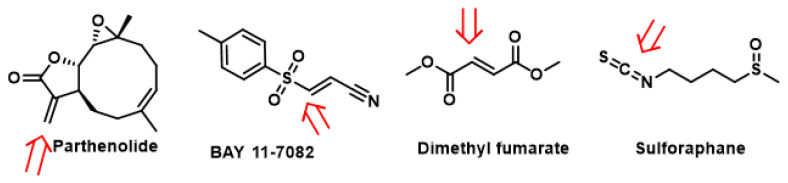
Molecular structures of parthenolide, BAY 11-7082, dimethyl fumarate (DMF) and sulforaphane. The red arrows indicate the point of attack.

- **Protection against irreversible damage**. Persulfidation safeguards the same hotspots from progression to sulfinic/sulfonic acid during oxidative bursts. Cells exposed to 100 µM H_2_O_2_ for 5 min accumulate 20–25% sulfinylated Cys38, but co-treatment with 1 µM polysulfides diverts > 80% of the modification to persulfide, which the thioredoxin system then clears within 10 min, restoring full DNA-binding capacity and curbing apoptotic signaling [53,166,167].
- **S-Alkylation as an emergency brake**. S-Alkylation functions as a rapid, irreversible brake on NF-κB signaling. Electrophilic inhibitors—such as the sesquiterpene lactone parthenolide, the vinyl sulfone BAY 11-7082, sulforaphane and dimethyl fumarate (DMF), (Figure 13), and the isothiocyanate sulforaphane—attack the critical Cys38/Cys179/Lys171 motif of RelA(p65) and IKKβ with second-order rate constants of approximately 10^2^ M^−1^ s^−1^—some 10^2^–10^3^-fold slower than protective persulfidation reactions [168,169]. These Michael additions generate stable C–S adducts that cannot be reversed by cellular glutathione. As a result, they produce (i): cys38 alkylation on RelA blocks its DNA-binding capability, and (ii) cys179 alkylation on IKKβ abolishes kinase activity and forms a reactive sulfonium, which can cross-link to Lys171, “locking” the activation loop in an open, inactive conformation [170,171].

Because these lesions are chemically irreversible, clearance of the modified proteins relies on proteasomal degradation rather than redox repair. The 20S/26S proteasome recognizes and eliminates the adducted IKKβ and RelA/p50 over the course of several hours, introducing a prolonged refractory period before NF-κB signaling can resume [172,173]. Under chronic electrophilic stress—or when clinicians exploit these agents therapeutically—S-alkylation thus acts as a pharmacological “kill switch”, but at the expense of widespread glutathione depletion (notably via DMF-mediated succination of Keap1 Cys151) and the risk of off-target alkylations across the proteome [155,174].

Kinetic, structural and cellular data place persulfidation as the primary, rapid-cycling rheostat that fine-tunes NF-κB signaling in response to fleeting redox cues, whereas S-alkylation remains a slower, more drastic countermeasure engaged only when persulfide buffering capacity is exhausted. Selective delivery of persulfide donors to inflamed tissue could therefore modulate cytokine output with temporal precision and fewer side effects than electrophilic inhibitors, while combined regimens might exploit the reversible-irreversible pairing to achieve both immediate damping and long-term suppression of pathological inflammation.

### 8.3. Selective Persulfidation and S-Alkylation of Reactive Cysteines in STAT3

Signal Transducer and Activator of Transcription 3 (STAT3) uniquely harbors a single hyper-reactive cysteine—Cys259—within its coiled-coil domain, which connects receptor-associated kinases to the SH2 dimerization module. Proteome-wide reactivity profiling has shown that Cys259 is among the rare cysteines with markedly elevated nucleophilicity, distinguishing STAT3 from other STAT family members [145,175]. Site-directed mass-spectrometric studies indicate that the intrinsic pK_a_ of Cys259 is depressed to approximately 7.4, such that at physiological pH (~7.4) roughly 25% of the side-chain thiols exist as the more reactive thiolate (–S^−^) form, priming this residue for rapid nucleophilic attack [176].

Two additional cysteines—Cys367, located in the DNA-binding domain, and Cys687, at the SH2/linker junction—exhibit lower baseline reactivity, yet both engage in redox cross-talk following modification of Cys259, thereby integrating multiple layers of redox regulation within STAT3’s activation cycle [177].

Under homeostatic conditions, Cys259 of STAT3 is rapidly persulfidated within seconds by rising levels of glutathione persulfide (GSSH) or low-molecular-weight polysulfides generated via the cystathionine γ-lyase/3-mercaptopyruvate sulfurtransferase pathways [178].

Stopped-flow kinetic analyses with recombinant STAT3 revealed a second-order rate constant of (4.5 ± 0.6) × 10^4^ M^−1^ s^−1^ for the reaction between Cys259 and glutathione persulfide (GSSH) at 25 °C. This rate is comparable to those of the fastest sensor cysteines in Keap1 and approximately 100-fold faster than rates measured for typical α,β-unsaturated carbonyl electrophiles. Structurally, the formation of the –SSH adduct on Cys259 extends the thiol side chain by approximately 1.8 Å and introduces a negatively charged terminus. This negatively charged group repels the acidic patch formed by Glu274 and Glu275, thereby disrupting the proper alignment of Tyr705 with the JAK2 active site and preventing phosphorylation-dependent dimerization of STAT3. At the same time, the presence of the additional sulfur atom destabilizes the proto-dimer interface at Leu257 and Leu261, promoting the transition of STAT3 into a monomeric and transcriptionally inactive state [178].

Clearance of Cys259 persulfidation is exceptionally rapid: the thioredoxin-1/glutaredoxin system reduces the –SSH adduct with a t_1_⁄_2_ of 20–25 s, restoring full STAT3 responsiveness once the redox signal subsides. Moreover, persulfidation protects Cys259 from irreversible over-oxidation or glutathionylation during oxidative bursts, enabling hundreds of redox cycles without permanent damage [179].

α,β-unsaturated carbonyls—including dimethyl fumarate, nitro-oleic acid and the small-molecule inhibitor STATTIC—alkylate Cys259 at 10^2^–10^3^ M^−1^ s^−1^, forging a covalent C–S bond that neither the Trx nor the Grx system can undo. The adduct perturbs the coiled-coil helix enough to preclude Tyr705 alignment for trans-phosphorylation and simultaneously traps STAT3 in a conformation incompetent for DNA binding. Because cellular clearance depends on proteasomal degradation, the inhibitory effect endures for 4–6 h, outlasting the ephemeral repression produced by persulfidation. Off-target liabilities emerge at the same doses, however, as the same electrophiles indiscriminately alkylate GAPDH, PARK7 and multiple mitochondrial enzymes, fuelling NAD(P)H depletion and secondary ROS accumulation.

Thus, STAT3 mirrors Keap1 and NF-κB in adopting a two-tiered redox logic: persulfidation operates as a rapid, self-limiting rheostat that attenuates signaling while preserving future responsiveness [180], whereas S-alkylation acts as a slower, emergency cut-off that silences the pathway at the price of forfeiting reversibility and risking collateral thiol loss. The kinetic supremacy of persulfidation suggests that endogenous RSS flux is the primary governor of STAT3 activity in physiological contexts such as cytokine resolution, mitochondrial biogenesis and stem-cell pluripotency [181]. Conversely, pharmacological alkylators gain therapeutic appeal in chronic oncogenic settings where prolonged STAT3 blockade is desirable, provided that delivery is tailored to minimise systemic thiol wastage [182]. Together, the structural, kinetic and cellular data argue for drug-design strategies that harness persulfide-releasing pro-drugs to fine-tune STAT3 in inflammatory or metabolic diseases, reserving irreversible electrophiles for oncology indications where sustained pathway suppression outweighs redox collateral [183].

## 9. Therapeutic Perspectives. Clinical Trials, Combined Strategy with Classic Antioxidants, Limitations and Analytical Challenges

### 9.1. Clinical Trials

The clinical translation of the ‘sulfur axis’ is underway. The following trials have been completed or are currently in progress (ClinicalTrials.gov, accessed on 2 September 2025).

In NCT02278276, the oral prodrug SG1002, which releases polysulfides in a sustained manner, completed a phase II in patients with heart failure: for 12 weeks it stably raised plasma H_2_S and nitrite and was associated with decreases in BNP and systolic blood pressure without serious adverse events, supporting the safety of the approach [18].

In NCT00879645, a phase I, single-center study assessed the pharmacokinetics and safety of IK-1001 (sodium sulfide) delivered as a 3-h IV infusion in 28 subjects stratified by renal function (healthy to severe impairment). Doses were 1.5 mg/kg/h for healthy and mild-to-moderate cohorts, and 1.0 mg/kg/h for severe impairment. Key measures included exhaled H_2_S, plasma/urine thiosulfate, and IK-1001 levels over 48 h, with a 7-day follow-up. The trial was terminated after completing recruitment due to challenges in developing a rapid, reliable sulfide assay.

NCT00858936 was a phase I/II, single-center trial assessing the safety, feasibility, and preliminary cardioprotective effects of a one-time, perioperative IV infusion of IK-1001 (liquid sodium sulfide) in patients undergoing elective coronary artery bypass graft (CABG) surgery. Up to six subjects received a weight-adjusted dose of IK-1001 immediately before graft reperfusion, with primary endpoints focused on safety/tolerability and exploratory biomarkers of myocardial injury (e.g., troponin, CK-MB). The study was terminated after enrolling six participants due to early sponsor decision, with no serious adverse events reported.

NCT01007461 was a planned phase IIa trial of IK-1001 (sodium sulfide, Na_2_S) for intravenous administration in patients with acute ST-segment elevation myocardial infarction (STEMI). The study aimed to evaluate safety, tolerability, and exploratory efficacy measures—such as myocardial salvage indices and cardiac biomarkers—following a single post-infarction infusion of Na_2_S. It was withdrawn prior to any participant enrollment due to a company decision unrelated to safety, with the record updated on 4 November 2010.

NCT01989208. Phase I, single-center trial evaluating safety, tolerability, and bioavailability of SG1002 in healthy volunteers (n = 7) and NYHA II–III heart failure patients (n = 8). Subjects received escalating doses of SG1002 (200 mg, 400 mg, then 800 mg), administered twice daily for 7 days each dose level. SG1002 was safe and well tolerated at all doses, produced dose-dependent increases in circulating H_2_S (notably at 400 mg) and nitrite (at 400 mg and 800 mg), and attenuated rises in BNP among heart failure subjects [18].

NCT01407172 was a completed, single-center observational study that enrolled 252 adults to evaluate plasma free H_2_S as a biomarker in peripheral arterial disease (PAD). Participants were stratified into no vascular disease, non-PAD vascular disease, and PAD groups. The primary outcome was plasma free H_2_S concentration, which was found to be significantly higher in PAD patients compared to controls (514.5 ± 62.1 vs. 368.5 ± 20.8 nmol/L; *p* = 0.007) [184].

NCT01232257 was a single-group, interventional study conducted from July to December 2011 at a single center in the Netherlands, designed to assess the effect of oral N-acetylcysteine (NAC) on plasma H_2_S levels and on markers of oxidative stress, inflammation, and endothelial dysfunction. Four cohorts were enrolled: healthy volunteers; patients with chronic kidney disease stage 3–4 (GFR 15–60 mL/min); hemodialysis patients; and peritoneal dialysis patients. All participants received NAC according to protocol, and the primary outcome measure was the change in plasma hydrogen sulfide concentration 48 h after dosing. The trial completed as planned, although no results have been posted.

NCT05457881 is a randomized, controlled, mechanistic trial sponsored by Brigham and Women’s Hospital (Boston, MA) to evaluate a short-term preoperative protein-calorie restriction (PCR) diet for endogenous H_2_S upregulation in patients undergoing elective lower-extremity arterial vein bypass surgery. A total of 226 participants were randomized 136:90 to a 4-day PCR intervention versus ad libitum diet. Primary endpoints include patient compliance, feasibility, and changes in plasma H_2_S levels and stress-related biomarkers before and after surgery.

NCT06095349 was a single-group, open-label interventional study assessing the effects of inhaled sulfureous thermal water aerosol on circulating H_2_S levels and bone metabolism markers in 50 postmenopausal women with osteopenia. Participants (aged 50–60) underwent 12 consecutive days of daily 30 min inhalation treatments. Primary outcomes were changes in bone turnover markers (P1NP, BAP, CTX-1) over 15 days; secondary outcomes included serum H_2_S levels measured by HPLC at the same timepoint. The trial was completed on 30 November 2023, with full enrollment and no safety concerns reported.

NCT06725797 is a pilot, single-center interventional study evaluating twice-daily immersion in H_2_S-rich sulfurous spa water for 14 days in adults with chronic musculoskeletal pain. Up to 40 participants receive standardized 20 min baths each morning and evening. Primary outcomes include change in pain intensity (VAS score) and quality-of-life measures over the treatment period; secondary outcomes assess plasma H_2_S levels and inflammatory biomarkers. The trial is currently in an unknown status with no results posted.

NCT05277285 is a phase II interventional trial evaluating the safety and tolerability of three escalating doses of intravenous sodium thiosulfate (STS) in mechanically ventilated, critically ill COVID-19 patients. The primary aim is to characterize STS safety profiles, with exploratory assessment of effects on oxygenation and inflammatory biomarkers in severe SARS-CoV-2–associated acute respiratory distress.

We refreshed trial registries and source publications as of 2 September 2025. Notably, the NAC study in CKD and dialysis cohorts (NCT01232257) remains listed as completed, with no results posted, indicating that quantitative outcomes are still unavailable to the community. In parallel, the early IK-1001 program (Na_2_S infusion) was curtailed by cross-platform analytical limitations—chiefly the inability to quantify sulfide reliably in vivo—which undermined exposure–response assessment and ultimately contributed to termination/withdrawal decisions in renal impairment, CABG and STEMI settings. These experiences underscore that future donor programs should anchor development plans to validated speciation analytics and standardized PK/PD readouts. Table 4 consolidates current status, reasons for termination or withdrawal where disclosed, and practical implications for next-generation sulfur therapeutics.

Collectively, these updates show that (i) analytical readiness was the pivotal failure point for first-generation IV donors (IK-1001), (ii) slow-release oral donors (SG1002) have achieved acceptable safety and bioactivity in early studies but still lack robust, assay-anchored phase II efficacy data, and (iii) biomarker and adjunct paradigms (e.g., PAD H_2_S, STS in STEMI) are informative yet remain heterogeneous in sampling and endpoints. Future trials should therefore be co-designed with validated speciation metrics and pre-agreed clinical endpoints to ensure reproducibility and interpretable dose–exposure–response mapping.

### 9.2. Combined Strategy

Controlled inhalation of H_2_S gas is moving from bench to bedside as an adjunct in SARS-CoV-2–induced respiratory-distress syndrome. In-vitro work with human airway and VeroE6 cultures showed that nanomolar (10–100 nM) H_2_S cuts viral titre by 1–2 log_10_, preserves NADH/NAD^+^ balance and sustains mitochondrial oxidative phosphorylation, largely through rapid activation of the Nrf2/Keap1 axis and repression of NF-κB-driven IL-6 output [112,188]. A brief report in Shock described five compassionate-use cases given humidified H_2_S at 20–40 ppm; no hemodynamic instability occurred and PaO_2_/FiO_2_ ratios trended upward, prompting calls for a formal trial [189]. That call has led to the phase-I/II “H_2_S-COV” protocol (NCT05277285), which delivers three daily 30 min sessions at 30 ppm to ventilated COVID-ARDS patients, focusing first on safety markers such as QTc, ionised Ca^2+^ and blood sulfide levels.

Mechanistically, inhaled H_2_S is expected to (i) block viral replication via NRF2-mediated antioxidant defence, (ii) stabilise mitochondrial membrane potential in hypoxic pneumocytes and (iii) dampen the cytokine surge by curbing IL-6 release and NET formation [188,190]. Co-administration of classic antioxidants may widen the redox safety window: intravenous vitamin C scavenges residual ROS, while N-acetyl-cysteine boosts endogenous H_2_S production and has already been shown to raise plasma sulfide in CKD and dialysis cohorts [191].

### 9.3. Limitations and Analytical Challenges

Key challenges remain. Alveolar concentrations above ~80 ppm drive blood H_2_S past ≈10 µM, the threshold at which cytochrome-c oxidase inhibition and sulfide toxicity appear; hence real-time amperometric monitoring is mandatory, accessed on 2 September 2025 [192]. Current bedside assays for free H_2_S or polysulfides still show coefficients of variation well above 20% because sulfur passivation erodes sensor sensitivity and common electro-chemical cartridges cross-react with SO_2_, NO_2_ and other acid gases [193] accessed on 2 September 2025. At the same time, medical-grade H_2_S lacks an official drug monograph in the European, U.S. or Japanese Pharmacopoeias—unlike oxygen or inhaled nitric oxide—leaving regulators without a consensus purity or impurity panel. Early clinical teams have therefore repurposed nitric-oxide delivery carts such as the INOmax DSIR-Plus, Mallinckrodt Pharmaceuticals, Dublin, Ireland, adding inline charcoal filters, over-pressure leak alarms and redundant flow monitors to mitigate accidental release while meeting GMP traceability, accessed on 2 September 2025. Until real-time amperometric analytics become more precise and a formal pharmacopeial standard is written, these technical and regulatory gaps will keep inhaled H_2_S in the “experimental adjunct” column—even as proof-of-concept studies urge its evaluation for viral ARDS and COVID-19-related respiratory failure [189].

The therapeutic margin is narrow: above ~10 µM free H_2_S inhibits cytochrome-c oxidase and flips from anti- to pro-oxidant [194,195], whereas below ~0.1 µM persulfidic signaling becomes negligible because endogenous H_2_S in most tissues sits in the low-nanomolar to sub-micromolar range. Keeping patients inside that “sweet spot” therefore demands controlled-release donors plus robust exposure biomarkers. Yet current bedside sensors show >20% coefficient of variation due to electrode passivation and cross-reactivity with other acid gases, and most clinical assays still report only “total blood sulfide”, which fails to distinguish free H_2_S from polysulfides or protein persulfides and ignores sub-cellular localization [39,196].

“Click-chemistry” persulfidomics has widened coverage but still under-quantifies long sulfur chains and lacks second-to-minute temporal resolution [196,197]. In-vivo imaging is likewise immature: PET probes such as [^64Cu^]ATSM show H_2_S-dependent uptake in animal models, but no tracer is yet validated for routine human use [198]. Finally, medical-grade H_2_S remains without a Pharmacopeia monograph, and occupational-exposure rules are still evolving, forcing clinical trials to rely on modified nitric-oxide delivery systems fitted with leak alarms and charcoal filters, such as the one developed by the U.S. Department of Labor (https://www.osha.gov/hydrogen-sulfide, accessed on 2 September 2025).

Until these analytical and regulatory gaps close, precise dosing and real-time monitoring will be the bottlenecks that keep inhaled or systemic sulfide therapy in the experimental realm.

Looking ahead to the next decade, priorities include (i) expanding phase II/III trials of oral and mitochondrial donors in heart failure and neurodegenerative disease: SG1002 has completed phase I/II trials showing safety and H_2_S/NO bioavailability in heart-failure patients (NCT02278276) [18,186], and mitochondria-targeted donors like AP39 are poised for first-in-human neurodegeneration studies following promising preclinical efficacy in Alzheimer’s models (Clinical trial NCT02278276); (ii) standardizing analytical panels that integrate persulfidation, free polysulfide, and redox potential: emerging “click-chemistry” persulfidomics workflows improve detection breadth but still under-quantify long sulfur chains and lack minute-scale temporal resolution, underscoring the need for harmonized, cross-platform standards (Clinical trial NCT022782); and (iii) testing rational combinations with NAC, vitamin C, or SGLT2-i in clinical outcome studies: N-acetyl-cysteine boosts endogenous H_2_S synthesis and improves redox markers in CKD cohorts, high-dose intravenous vitamin C scavenges residual ROS in septic and ARDS patients, and adjunctive SG1002 plus SGLT2 inhibition has shown additive benefit in HFpEF models, prompting phase II/III combination trials. Only in this way can the biochemical versatility of sulfur be converted into safe and effective medicines that complement the classic antioxidant artillery.

### 9.4. Safety, Contraindications and Drug–Drug Interactions

Translational programs converge on a narrow physiological window for sulfide: sustained free H_2_S above ~10 µM can inhibit cytochrome-c oxidase and flip signaling from protective to toxic. In inhaled approaches, alveolar concentrations >~80 ppm risk pushing blood sulfide past this threshold, hence the emphasis on real-time monitoring. These constraints should guide dosing, monitoring and stopping rules across platforms.

In phase I/II settings SG1002 increased circulating H_2_S/nitrite and attenuated BNP without serious adverse events, supporting acceptable short-term tolerability. Common anticipated reactions are mild hypotension or dizziness in dose-escalation phases; long-term safety still requires larger trials. Contraindications/caution: uncontrolled hypotension, brittle heart failure with narrow perfusion reserve. Interactions: additive blood-pressure lowering with high-dose nitrates or PDE-5 inhibitors—separate dosing by several hours.

Early peri-operative/renal-function studies focused on safety/PK and serious adverse events were uncommon before early terminations for non-safety reasons. Nevertheless, fast peaks are non-physiological and more likely to provoke hypotension and transient respiratory-chain inhibition if exposure exceeds the safe window; their clinical path remains limited.

Sodium thiosulfate (STS) was explored mainly for safety/tolerability in critical-care settings. Specific cautions: transient calcium chelation—monitor ionised Ca^2+^ and QTc, especially in renal impairment or when combined with QT-active drugs.

In short protocols (e.g., 30 ppm sessions), inhaled sulfurous therapies have shown physiological improvements without hemodynamic instability in early experiences; however, delivery must include gas-analytics and interference-tested sensors, given cross-reactivity and passivation issues. Until pharmacopoeial standards exist, these remain investigational and require controlled environments. Contraindications are severe reactive airway disease, inability to comply with monitored delivery.

As “smart”/mitochondria-targeted donors (AP39, photo/thiol-activated scaffolds), preclinical benefits are promising, but human safety data are lacking; off-target mitochondrial effects at high local concentrations remain a theoretical risk, arguing for conservative first-in-human dose-finding.

In dietary sulfur compounds (garlic polysulfides; cruciferous isothiocyanates), animal and ex-vivo data support cardiometabolic and vascular benefits mediated by H_2_S release; clinical interaction profiles are not yet defined. We recommend explicit reporting of culinary processing and background diet/microbiota in trials, as these factors modulate bioavailability and hence exposure.

Sulfide production in the colon depends on diet and community composition, and microbiota interventions may shift luminal sulfide outside the protective range in susceptible hosts; monitoring of symptoms and redox markers is advisable in proof-of-concept studies.

Cross-platform interactions and co-therapies in (i) vasoactive combinations: avoid stacking with potent nitrates or PDE-5 inhibitors without spacing; watch for symptomatic hypotension. (ii) antioxidant co-therapy: NAC can raise endogenous H_2_S and is a plausible add-on; titrate empirically. (iii) analytics-driven care: where possible, anchor titration to circulating H_2_S/derivatives and routine chemistry to remain within the 0.3–5 µM corridor suggested by early translational work.

Across platforms, the principal risks trace back to dose kinetics (spikes vs. sustained release) and measurement uncertainty (gas delivery and biosensor bias). Short-term safety signals exist for SG1002 and monitored inhalation, robust data on long-term outcomes, rare adverse events, and formal interaction studies are still needed before routine clinical use.

## 10. Combination and Sequential Therapies Based on Reactive Sulfur Species/Persulfidation

Clinical studies reveal a time-layered sulfur physiology. The trials tell a coherent story: each therapeutic modality delivers H_2_S or other RSS for only a limited slice of the illness timeline, so no single agent can cover the whole arc from the first hypoxic hit to long-term tissue remodelling [195]. This section therefore sets out to match the right tool to the right time-window, stitching them together into a relay that keeps persulfidation inside the protective 0.3–5 µM range and steers clear of the >10 µM toxicity zone:

- Intravenous “flash” donors such as IK-1001 (Na_2_S) deliver a sharp H_2_S/RSS peak lasting only a few hours—useful in peri-ischemic settings but unsuited to longer phases (Clinical trial NCT00858936).

Oral slow-release pro-drugs (e.g., SG1002) climb more slowly yet sustain micromolar H_2_S for days-to-weeks [18] (Clinical trial NCT0227827).

Dietary, microbiome or balneotherapy approaches can keep a low-level RSS “tone” for months and are now in formal trials (NCT05457881, NCT06095349).

### 10.1. Condensed Clinical Roadmap

Table 5 functions as a condensed clinical roadmap. By aligning the three illness phases—0–24 h “flash” rescue, Day 2–14 catalytic maintenance, and ≥Week 3 chronic remodelling—with their dominant sulfur target, prototype intervention and the human trial that supports each move, the table shows at a glance when to hand the baton from an intravenous donor to a slow-release oral drug and finally to diet- or microbiome-based measures. In short, it turns scattered evidence into a time-layered decision aid that keeps persulfidation within the 0.3–5 µM safety window while avoiding therapeutic gaps or overlaps:

### 10.2. Clinical Sequence

**Figure 14:**
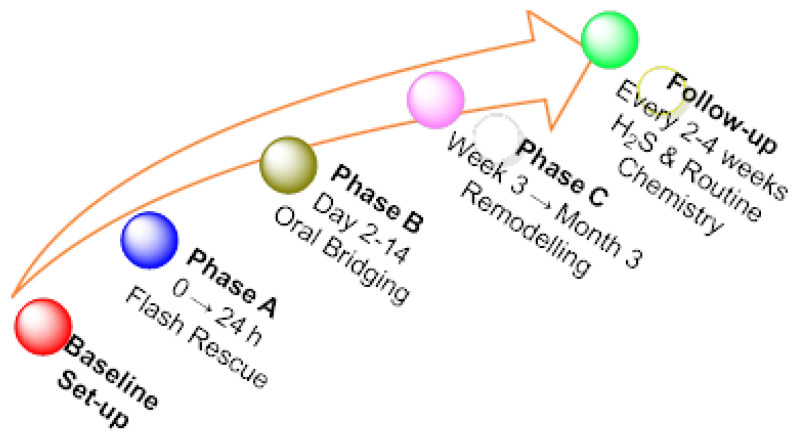
Time-stamped clinical sequence.

This subsection lays out a time-stamped clinical sequence, Figure 14—starting with a baseline set-up (labs, ECG/echo, central line), moving through 0–24 h intravenous “flash rescue”, Day 2–14 oral bridging, and Week 3 → Month 3 lifestyle-based remodelling—then rolls into a follow-up phase in which H_2_S or, ideally, protein persulfidation plus routine chemistry are checked every 2–4 weeks.

The aim is to give front-line teams a ready-to-use roadmap that aligns each therapeutic layer with the patient’s evolving redox biology, tells them exactly when to hand the baton from one intervention to the next, and builds in laboratory checkpoints to keep dosing inside the 0.3–5 µM safety corridor while avoiding gaps or overlaps in coverage:

**1—Baseline set-up** (≈1 h before starting any donor).

Start with a one-off bundle: draw full labs (including lactate, creatinine and—if the lab can do it—free H_2_S or a persulfidation surrogate), perform a 12-lead ECG and focused bedside echo, and rule out G-6-PD deficiency. A central line is advisable because both candidate i.v. donors are acidic and need controlled infusion rates [199].

**2—Phase A**—Intravenous “rescue” (T 0 → 24 h).

a. IK-1001 (injectable Na_2_S)—The first-in-human coronary-bypass study escalated single boluses from 0.005 to 0.1 mg kg^−1^ with no clinically significant adverse events. That trial was halted only because bedside sulfide analytics were still unreliable, not for safety concerns [200]. A practical rescue schedule is a short infusion delivering ≤0.1 mg kg^−1^ over three hours immediately after reperfusion or cardiac arrest.

b. Sodium thiosulfate (STS)—The open-label H4COVID trial (NCT05277285) is giving three i.v. doses of STS to ventilated COVID-ARDS patients to characterise safety and look for clinical benefit. Mimicking that protocol (e.g., a 6 g bolus followed by two 4 g doses 12 h apart) is reasonable for sepsis or non-COVID ARDS, provided ionised calcium and QTc are watched.

Stop criteria for either donor: MAP < 55 mmHg unresponsive to fluids, SpO_2_ < 88% on FiO_2_ 1.0, arrhythmia, or plasma H_2_S climbing above ~10 µM (the level at which Complex IV inhibition becomes appreciable)

**3—Phase B**—Oral “bridging and consolidation” (Day 2 → 14).

SG1002 slow-release tablets—In a phase-I/II trial in heart-failure patients, twice-daily doses of 200 → 400 → 800 mg raised circulating H_2_S and nitrite while blunting BNP, with an excellent safety profile [18]. A common clinical tactic is to start at 400 mg BID once the patient can swallow, check plasma H_2_S after 48 h, and up-titrate to 800 mg BID if levels remain below ~0.3 µM.

Adjunct N-acetyl-cysteine (NAC)—A completed study in CKD and dialysis patients showed NAC supplementation increases endogenous H_2_S and improves redox markers, making it a logical add-on when oxidative burden is high or when tapering donors.

**4—Phase C**—Long-term remodelling (Week 3 → Month 3).

Dietary module—A four-day protein-calorie-restriction (PCR) diet immediately before major vascular surgery is being tested in a 226-patient RCT (NCT05457881) specifically to boost endogenous H_2_S. After PCR, switch to a Mediterranean pattern rich in garlic and cruciferous vegetables (both cysteine- and isothiocyanate-rich).

Balneotherapy/aerosol module—The FORST-3 study (NCT06095349) found that 12 consecutive days of inhaling high-sulfide spa water safely lifted systemic H_2_S in osteopenic women. For chronic wounds or neuropathic pain, a pilot trial using hypothermal sulfurous water immersion is open (NCT06725797).

**5—Follow** **-up and titration.**

Re-measure H_2_S (or, ideally, protein persulfidation) plus routine chemistry every 2–4 weeks:

- If H_2_S < 0.3 µM → increase SG1002 by 200 mg steps (max 1 g BID).
- If H_2_S 5–10 µM and lactate steady → maintain.
- If H_2_S > 10 µM or lactate rises → halve or hold one dose and re-check in 48 h [195].

**6—Safety corridor and drug** **-interaction notes.**

- Sustained free H_2_S above ~10 µM blocks cytochrome-c oxidase and flips the gas from cytoprotective to cytotoxic.
- STS can transiently chelate calcium; check ionised Ca^2+^ and QTc in renally impaired patients.
- Combining any sulfide donor with high-dose nitrates or PDE-5 inhibitors may amplify hypotension—space administrations by several hours.

**7—Putting it all together.**

Think of the plan as a relay race:

- Sprinter—a brief i.v. sulfide or thiosulfate surge buys time during the first 24 h of ischemia–reperfusion or fulminant inflammation.
- Middle-distance runner—slow-release SG1002 (±NAC) keeps micromolar persulfidation humming through the sub-acute week.
- Marathoner—diet and sulfur-rich spa interventions retrain endogenous and microbiome sulfur metabolism for the long haul.

Hand the baton only when the next runner is up to speed and you stay within that narrow 0.3–5 µM plasma window. Early human trials suggest this choreography can deliver redox protection without crossing the toxicity line, but every step remains investigational and should live inside a monitored research protocol.

## 11. Conclusions

The evidence reviewed establishes RSS and protein persulfidation as a third redox axis on par with oxygen and nitrogen, distinguished by its exceptional chemical plasticity. Endogenous H_2_S production—via CBS, CSE and 3-MST—delivers a nanomolar “drip” that, upon oxidation by sulfide–quinone oxidoreductase, generates persulfides and polysulfides. These species funnel electrons into the respiratory chain with minimal ROS cost, quench oxidative bursts, and install reversible –SSH marks on key cysteines (e.g., Keap1, GAPDH, p47^phox^, caspase-1, eNOS), thereby coupling energy metabolism, vascular tone, inflammasome control and neuroplasticity.

When the sulfur “window” narrows—due to aging, dysbiosis or enzyme defects—pathologies from heart failure and sarcopenia to neurodegeneration, cancer progression and post-COVID sequelae surge. Restoring this window is now feasible through a layered therapeutic toolkit:

- Slow-release oral donors (SG1002, GYY4137) sustain safe H_2_S elevations.
- Organelle-targeted vectors (AP39, gem-dithiol pro-drugs) fine-tune delivery within mitochondria.
- Multi-gas hybrids synergize sulfur with NO or CO.
- Dietary interventions (garlic polysulfides, cruciferous isothiocyanates) and microbiota engineering reinforce endogenous RSS tone.

Key challenges ahead are twofold: (1) developing real-time analytics capable of discriminating free H_2_S, polysulfides and subcellular persulfidation; and (2) defining dose ranges that maximize protection without breaching the ~10 µM complex IV-inhibition threshold. Overcoming these hurdles will transform RSS into a transversal pharmacological platform, rebalancing redox and metabolic homeostasis across the era’s major chronic diseases.

Over the next 2–3 years, progress should follow a focused, three-strand roadmap. First, fit-for-purpose analytics and inter-lab standards: establish community SOPs for sampling and derivatization; adopt a reference tag-switch workflow with isotopic quantification; and implement benchmarking of sulfane–sulfur probes plus QA for real-time sensors, so that speciation (free H_2_S vs. polysulfides vs. protein-SSH) is stabilized and coefficients of variation are reduced. Primary stakeholders include analytical chemists and proteomics cores, clinical chemistry laboratories, instrument/bioprobe vendors, and—crucially—journal editors and method consortia to mandate reporting checklists. Second, dose–exposure–response mapping and a safety corridor: define platform-specific PK/PD and kinetic windows (slow vs. rapid releasers; organelle-targeted vectors), anchored to validated H_2_S/SSH readouts to preserve efficacy while avoiding complex-IV inhibition; key actors are clinical pharmacologists, early-phase trialists and DSMBs, donor developers, and regulators. Third, indication-specific clinical validation and endpoints: harmonize endpoints and staging across pilot trials (including time-layered combinations and sequential strategies), integrating the analytics from the first strand to prove target engagement and guide hand-offs between modalities; here the primary stakeholders are disease-area consortia and trial networks, biostatisticians, patient groups, and payers. Taken together, this roadmap converts methodological progress into a practical translational pipeline with clear ownership and milestones.

## Figures and Tables

**Table 1:** Main features of Reactive Species.

Property	ROS	RNS	RHS	RSS
Central atom	O	N	Cl/Br/I	S
Canonical examples	O_2_^•−^, H_2_O_2_, ^•^OH	^•^NO, ONOO^−^, NO_2_^•^	HOCl, HOBr, Cl_2_^•−^	H_2_S, RSSH, S_4_^2−^
Half-life	ns–ms (^•^OH) a ms–s (H_2_O_2_)	µs–ms (ONOO^−^)	μs (HOCl)	MS–min (polysulfides)
Biosynthetic pathways	Mitochondria, NADPH oxidase	US + ROS	MPO + halide	CBS/CSE, MPST
Prevailing changes	Oxidation (-OH, =O)	Nitro-/nitrous-	Chlor(in)ation, bromination	Persulfidation (-SSH)
Physiological functions	Signaling, immunity	Vasorrelaxation, defense	Microbicide inflammation	Bioenergy, mitochondrial protection

**Table 2:** Representative second-order rate constants (k_2_) for persulfide formation and H_2_S/HS^−^ oxidation.

Process/Reaction (Representative)	k_2_ (M^−1^ s^−1^)	pH (BUFFER)	T (°C)	Notes
HS^−^ + protein–SOH → protein–SSH (Mt AhpE–SOH model)	1.4 × 10^3^	~7.4 (pH-independent after correction)	n.r.	Nucleophilic attack of HS^−^ on sulfenic acid
Hydrophilic LMW-persulfide + protein-Cys → protein–SSH	10^4^–10^5^	~7–7.5	n.r.	Stopped-flow; faster than Michael additions
Michael electrophile + reactive Cys (comparative)	~10^2^	~7.4	n.r.	Irreversible C–S adduct formation; comparator scale
H_2_S + H_2_O_2_ → HSOH → (polysulfides/S^0^/SO_4_^2−^)	0.73	7.4	37	Product distribution depends on [H_2_O_2_]:[H_2_S]
HS^−^ + ONOOH → HSOH + NO_2_^−^ (±HSSH)	6.7 × 10^3^	7.4	37	Nucleophilic substitution pathway
HS^−^ + HOCl → HSCl → HSOH (rapid hydrolysis)	0.8–20 × 10^8^	7.4	n.r.	Among the fastest oxidations of HS^−^
HS^−^ + taurine-chloramine → oxidized products	3 × 10^2^	7.4	37	Slower than HOCl; higher selectivity

Abbreviations: LMW, low-molecular-weight; n.r., not reported in source. Values are apparent under the specified conditions and may vary with ionic strength, buffer and matrix; they are provided here as practical ranges to frame Section 2 and Section 4.

**Table 3:** Rate constants determined for reactions of H_2_O_2_, ONOOH and HOCl with H_2_S.

Oxidizing	k_2_ M^−1^ s^−1^	pH	Temperature	Reference
Hydrogen peroxide	0.73	7.4	37 °C	[59]
Peroxynitrous acid	6.7 × 10^3^	7.4	37 °C	[60]
Hypochlorous acid	(0.8–20) × 10^8^	7.4		[61]
Taurine chloramine	3 × 10^2^	7.4	37 °C	[41]

**Table 4:** Updated status of human studies targeting the sulfur axis (as of 2 Sep 2025).

NCT ID	Agent/ Modality	Indication and Design	Phase	Current Status	Results/Reference	Reason for Termination/Note	Practical Implication
**NCT00879645**	IK-1001 (sodium sulfide, IV)	PK/renal function stratification, single 3-h infusion	I	Terminated	No posted results [185]	Program hit a core analytical barrier: inability to reliably determine sulfide levels in vivo	Future IV “flash” donors require validated, rapid speciation assays before dose-escalation.
**NCT00858936**	IK-1001 (IV)	Peri-operative CABG, single pre-reperfusion dose	I/II	Terminated	No posted results [185]	Reasons not reported publicly; part of broader assay/measurement challenges	De-risk peri-ischemic studies by pre-specifying orthogonal exposure readouts (e.g., thiosulfate, bound sulfane sulfur).
**NCT01007461**	IK-1001 (IV)	STEMI, post-infarction infusion	IIa	Withdrawn	[185]	Company decision (non-safety-related)	Confirms strategic, not toxicity-driven, halt; emphasizes the need for assay-ready platforms.
**NCT01989208**	SG1002 (oral slow-release H_2_S donor)	HF patients; dose-escalation, 200–800 mg BID	I (HF)	Completed	Published [18]	—	Supports feasibility of sustained donors; motivates phase II designs with standardized endpoints.
**NCT02278276**	SG1002 (oral)	Heart failure; randomized, placebo-controlled	I/II	Status: unknown/no results posted (record not recently updated)	[186]	No public readout	Consolidate HF endpoints and ensure assay-anchored target engagement in future trials
**NCT01407172**	Biomarker study (plasma free H_2_S)	Peripheral arterial disease; observational	—	Completed	Published [184]	—	Demonstrates clinical biomarker feasibility but highlights pre-analytical sensitivity; standardization needed
**NCT01232257**	N-acetylcysteine (NAC) (endogenous H_2_S modulator)	4 cohorts (healthy, CKD 3–4, HD, PD); single-group	—	Completed (2011)	No posted results	—	Keep as background evidence of feasibility; pursue modern speciation and kinetic sampling in any replication.
**NCT02899364**	Sodium thiosulfate (STS) (indirect H_2_S donor)	STEMI, proof-of-principle	II	Completed	Published [187]	—	STS remains attractive for safety; refine timing, dose, and analytics to capture cardioprotection signal.

NCT01232257 (NAC): registry indicates Completed with no results posted; no peer-reviewed outcome paper identified; IK-1001 program: the measurement problem (inability to quantify sulfide reliably) is a central limitation and linked to the renal-impairment PK study termination; CABG trial was terminated after only six participants; the STEMI trial was withdrawn by company decision (non-safety) [185]; SG1002 phase I HF readout (dose-dependent ↑H_2_S/nitrite, good tolerability) is published; the subsequent phase I/II record shows unknown/no posted results [18]; PAD observational study provides a quantitative H_2_S biomarker dataset (JAHA 2013) [184]; STEMI STS: recent proof-of-principle results published in 2023 [187].

**Table 5:** Clinical roadmap.

Layer	Time-Window	Main Objective	Prototype Interventions	Key Clinical Evidence
Rescue (“flash”)	0–24 h around infarction, CABG, early ARDS	Rapid mitochondrial rescue/IPC mimicry	IV IK-1001 1.0–1.5 mg kg^−1^ h^−1^ × 3 h; IV sodium thiosulfate 6 g bolus → 4 g q12h	IK-1001 tested in CABG (n = 6) with no serious AEs, programme halted for analytical—not safety—issues; STS dose-escalation in ventilated COVID-ARDS is under way (NCT05277285)
Sustained catalytic	Day 2 → 14	Maintain systemic persulfidation	Oral SG1002 400–800 mg BID (phase I/II); GYY4137 slow-donor (pre-clinical)	SG1002 raised plasma H_2_S and nitrite and lowered BNP in heart-failure patients without safety signals (NCT02278276)
Chronic remodelling	≥3 weeks (rehab/long-COVID/CVD risk)	Re-educate endogenous and microbiome sulfur routes	Protein-calorie restriction (PCR) diet 4 d pre-surgery; H_2_S thermal-water aerosol 12 d; Sulfurous spa immersion 14 d; High-allium and crucifer diet ± pro-/pre-biotics	PCR trial in vascular surgery (n = 226) ongoing (NCT05457881); aerosol H_2_S raised circulating levels without safety issues (NCT06095349); spa immersion pilot recruiting (NCT06725797)
